# Effect of food variety on intake of a meal: a systematic review and meta-analysis

**DOI:** 10.1093/ajcn/nqaa352

**Published:** 2021-01-29

**Authors:** Rochelle Embling, Aimee E Pink, Jennifer Gatzemeier, Menna Price, Michelle D Lee, Laura L Wilkinson

**Affiliations:** Department of Psychology, College of Human and Health Sciences, Swansea University, Swansea, UK; Department of Psychology, College of Human and Health Sciences, Swansea University, Swansea, UK; School of Social Sciences, Nanyang Technological University, Singapore, Singapore; Department of Psychology, College of Human and Health Sciences, Swansea University, Swansea, UK; Department of Psychology, College of Human and Health Sciences, Swansea University, Swansea, UK; Department of Psychology, College of Human and Health Sciences, Swansea University, Swansea, UK; Department of Psychology, College of Human and Health Sciences, Swansea University, Swansea, UK

**Keywords:** food variety, intake, systematic review, meta-analysis, dietary diversity

## Abstract

**Background:**

Many studies have shown that food variety—the presence of multiple foods and/or sensory characteristics within and across meals—increases intake. However, studies report mixed findings, and effect size remains unclear.

**Objectives:**

A systematic review and meta-analysis were conducted to *1*) synthesize data across experimental studies that examined effects of variety on total meal intake, relative to a control condition with comparatively less variety; *2*) quantify support for this effect; and *3*) assist in the identification of important moderating factors (registration: CRD42019153585).

**Methods:**

In November 2019, we searched the following databases for relevant experimental studies, published in English from 1980, with human participants: PubMed, Cochrane Library, Web of Science, ClinicalTrials.gov, PsycINFO, and OpenGrey. This search was updated in September 2020. Means, standard deviations, and sample sizes were extracted from included articles, and Hedges' *g* was used to calculate effect sizes. Risk of bias was assessed using the Cochrane Collaboration's tool.

**Results:**

Of 7259 references identified in an initial search, 34 articles consisting of 37 studies contained sufficient information for review, and data from 30 studies (39 comparisons) were included in the meta-analysis. Results from a random-effects model showed a significant small to medium effect of variety on intake (in weight and energy), with greater variety being associated with increased consumption (Hedges' *g* = 0.405; 95% CI: 0.259, 0.552). However, heterogeneity was considerable across studies (*I*^2^ = 84%), and this was unexplained by subgroup analyses based on form of variety, test foods, sensory characteristics, age, sex, and body weight.

**Conclusions:**

Our findings support the conclusion that variety is a robust driver of food intake. However, risk of bias was high across studies, and this review highlights methodologic limitations of studies. It is recommended that further attention is given to the development of preregistered, well-powered randomized controlled studies in eating behavior research.

## Introduction

Eating a variety of foods—in a single meal or across days—is one factor that has been shown to increase food intake (1–4), with seminal experimental research on this effect (in human participants) being published by Barbara Rolls and colleagues in 1981 (1). As “food variety” is generally considered a good indicator of nutritional adequacy, this effect of variety can be beneficial to diet quality (5). For example, studies have explored how variety can be used to promote intake of fruit and vegetables in children (6), as well as encourage greater intake in older adults to combat undernutrition resulting from decreased appetite (7). However, variety may also increase the risk of overeating when foods are energy dense. Indeed, there is growing evidence to suggest that, excluding fruits and vegetables, greater dietary variety increases body weight and body fatness (8, 9). Given that both obesity and undernutrition continue to be global health concerns (10, 11), it is important to consider the influence of variety on intake.

The effect of variety on intake is generally thought to operate via the mechanism of sensory-specific satiety, the decline in the palatability of a food as it is consumed relative to uneaten foods that differ in sensory qualities (12). For example, Hetherington et al. (13) interrupted a meal to ask participants to taste-test a different, similar, or identical food (to that of the eaten meal); participants rated the eaten food more favorably and intake was greater when foods with a different or similar taste were presented.

In the current literature, most experimental studies have focused on the presence of variety within meals (where foods are presented in single or multiple courses at one sitting) and across meals (where foods are presented in meals across multiple sittings, in a single day, or several days) (14). These studies typically vary the number of different foods or sensory characteristics (i.e., appearance, tastes, textures, smells) present in meals and examine effects on ad libitum food intake in the laboratory.

Multiple narrative reviews have reported on the effects of variety on intake (14–16). These reviews conclude that the effect of variety on intake appears to be robust and is relatively consistent across studies. However, these reviews have also identified potential moderating factors. For example, some studies fail to find an effect when variety is manipulated for low-energy foods (17, 18) and when only a single sensory characteristic is varied (2, 19). The effect of variety also appears to be smaller in older adults (20), and some studies have failed to find an effect in males (21, 22). This means that the size of the effect of variety on intake has remained unclear.

Therefore, in this systematic review and meta-analysis, we examined the effect of variety within and across meals on intake to formally synthesize data across experimental studies. We report findings on the size of the effect of variety on total meal intake (in weight and energy) and assess differences in this effect when potential moderators are considered via subgroup analyses. For this review, we view “food variety” as a continuous metric, so that a “high-variety” condition has a comparatively greater number of components and/or sensory characteristics than a “low-variety” control condition (from our conceptual viewpoint).

## Methods

### Search strategy

We searched 6 electronic databases during November and December 2019: PubMed, Cochrane Library, Web of Science, ClinicalTrials.gov, PsycINFO, and OpenGrey. Searches used a combination of key terms relating to food variety and intake (see **Supplementary Methods 1**), as well as the period of consumption (i.e., specific mealtimes). All searches were limited to include studies that were published in English, with human participants, between January 1980 and the date of the search, in line with when seminal research on this effect was conducted (1). One author conducted the formal search of electronic databases (RE). Two authors independently screened and selected articles for review using titles and abstracts, as well as independently assessed full texts for eligibility (RE and AEP). Any disagreements were discussed and revised accordingly, and if needed, a third author was available to resolve outstanding discrepancies (LLW). Agreement rate was not recorded, as conflicts were discussed at intervals.

To reduce risk of publication bias, an invitation to provide any relevant published or unpublished work was shared with corresponding authors of included articles, posted on social media, and circulated via mailing lists of groups with relevant research interests. The database search was updated to include any potential new articles in September 2020, and a hand-search was also conducted in June 2020; reference lists of included articles were scanned for eligible studies, and relevant peer-reviewed journals were searched for articles published after the date of our initial search.

This systematic review and meta-analysis is reported in line with Preferred Reporting Items for Systematic Reviews and Meta-Analysis guidelines and was preregistered in the PROSPERO database (International Prospective Register of Systematic Reviews; registration number: CRD42019153585). The web-based software “Covidence” was used to manage and screen references (https://www.covidence.org).

### Study eligibility criteria

Experimental, quasi-experimental, or intervention studies, using either a within- or between-subjects design, were eligible. There were no restrictions on the age of participants included in studies or weight status. Studies were included if they manipulated the level of variety within or across meals and if they assessed how much food participants subsequently consumed in weight, energy, or number of pieces. Studies were required to have at least 2 experimental conditions consisting of a high variety, a comparatively lower variety, and/or a no-variety condition. This could include manipulations of the number of different foods presented or the number of different sensory characteristics included within a single food item or assortment. Studies that involved participants with chronic diseases, illnesses, or eating disorders; used only measures of food choice, food ratings, portion size selection, perceived volume, or self-reported intake (e.g., food diaries and questionnaires); and manipulated only the perception of food variety (e.g., by changing the presentation of foods but not the components present in the meal or by using labels/vignettes) were excluded in order to focus on effects on actual intake [pertaining to seminal research on the variety effect (1)].

Studies that were designed to measure sensory-specific satiety were included only if they had a comparator condition and the measurement of food intake was not disrupted (e.g., by asking participants to provide ratings of liking/satiation by tasting samples midway through a meal). Studies that otherwise met our inclusion criteria but asked participants to “earn” food servings as part of a habituation task were excluded, because food intake was disrupted by the need to gain points [e.g., (23)]. Relevant studies that were designed to measure early food acceptance in young children and infants were also excluded [e.g., (24, 25)], as foods in both the variety and comparator conditions were repeatedly presented to participants over a series of trials.

### Data extraction

One author extracted information from all eligible studies for review (RE), and a second author checked that the extracted data were consistent with information reported in articles (JG). Two authors also independently extracted information from 20% of included studies (RE and JG); the initial agreement rate was 82%, all conflicts were resolved after discussion, and extracted information from all articles was again checked for consistency. Where necessary, we contacted corresponding authors to provide missing data required for the meta-analysis (e.g., means, SDs, sample size), and data were extracted from figures using the online program “WebPlotDigitizer” (https://apps.automeris.io/wpd/). If standard errors and means were reported, standard deviations were calculated [SEM * sqrt(*N*)]. In order to allow for comparisons across studies, we focused on effects on total meal intake (all components of a meal), as analyses of individual components (e.g., single food within a course consisting of multiple foods) differed greatly between articles. See **Table 1** for a list of extracted information.

**TABLE 1:** List of information extracted from included articles for each study.

Information	Description
Sample characteristics	Including the country where the study was conducted, sample size, age, sex, and BMI
Study design	Between-subjects or within-subjects
Study setting	Laboratory or field
Study intervention	The form of variety manipulated, the number of sensory characteristics manipulated, and the comparator condition(s) used
Study outcome	Procedure to measure food intake, test foods eaten, and unit of measurement reported
Main findings	Results reported for main effect of variety on total food intake and, if available, individual components of a meal
Moderating variables	Interactive effects of identified moderators on food intake
Quality assessment	Including assignment to conditions, control procedures prior to manipulation, measurement and control of appetite and food ratings, additional variables controlled in data analyses

### Meta-analysis

All analyses were calculated using the software “Comprehensive Meta-Analysis v3” (Biostat). We used a random-effects model for all analyses, as the design, quality, measures, and samples used differed across studies. For each identified comparison in the main analysis, we inputted data for effects on total meal intake and used sample sizes, means, and standard deviations to calculate effect size. Hedges' *g* is reported, as it uses the standardized mean difference to account for the reporting of different units of intake across studies, corrects bias associated with very small samples (26), and has been used in a similar meta-analysis in which food intake was the outcome (27). Where studies reported intake in both energy and weight, we extracted information on weight consumed only. For all studies, a positive effect size indicates that food intake was greater in the high-variety condition relative to the control condition, whereas a negative effect size indicates that food intake was greater in the control condition relative to the high-variety condition. Effect sizes were interpreted as <0.2 is trivial, 0.2 is small, 0.5 is medium, and 0.8 is large (28). Heterogeneity was assessed using the *I*^2^ statistic and, as an approximate guide, was interpreted as <30% is low heterogeneity, 30–50% is moderate heterogeneity, 50–75% is substantial heterogeneity, and >75% is considerable heterogeneity across studies (29).

Exploratory sensitivity analyses were conducted to check for potential issues with study inclusion; we conducted a “1 study removed” analysis and reran analyses with adjusted imputed values for missing data. Planned subgroup analyses were conducted to investigate potential moderating effects of the form of variety manipulated, the number of sensory characteristics varied, the test foods used, and participant demographics (age, sex, and body weight). To account for multiple comparisons, Bonferroni-corrected *P* values are also reported.

To calculate effect sizes in which multiple datapoints within a study referred to the same participant, we contacted authors to provide the correlation between conditions for each comparison. Authors of 8 studies were able to provide this information, and we imputed the mean for all other studies after conducting relevant sensitivity analyses (*r* = 0.67) (see exploratory analyses described above for details of method of imputation). Where necessary, we computed a single summary effect for each study reporting multiple comparisons for the same participants by calculating the mean effect size and variance of the mean effect size (30). In this case, this meant that for studies reporting separate comparisons for single-food control conditions (e.g., variety compared with apple, variety compared with pear) and where effects for more than 2 levels of variety were reported, control conditions were combined to form a single comparison group (i.e., high variety compared with single foods; high variety compared with medium and low variety).

### Risk of bias

Two authors assessed risk of bias using the Cochrane Collaboration's tool (RE and AEP) (31), and disagreements were resolved in discussions. Following Buckland et al. (27), we considered risks associated with the following: participant and experimenter awareness of the study aim and assignment to conditions, the control of confounding variables that could influence food intake, the use of procedures to control for baseline differences in appetite, the use of procedures to measure food intake, and the order of tasks in study protocols (see **Supplementary Methods 2** for details on assessment criteria).

## Results

Unless otherwise stated, mean ± SD is reported. See **Table 2** for a description of each study eligible for review.

**TABLE 2:** Characteristics of studies included for review (*N* = 37)^1^

First author (year), country^2^	Design and participants	Form of variety, test food, and outcome measure	Main result for total food intake (where relevant, without beverages)	Additional results for food intake (a single or combination of components within a meal)	Interaction effects and/or subgroup analyses for food intake	Methodological considerations
Appleton (2009), UK (32)	Field (nursing home); within-subjects; order of conditions counterbalanced across nursing homes, but method is unclear. Sample = 28 older adults (21 females).Age range = 65+ y.BMI = not reported. No exclusions	Variety manipulated within a single course; sauces were provided with a main meal of the day consisting of a meat, 2 vegetables, and potatoes. In each condition for each nursing home, participants were served 2 meals (usual portion sizes presented for each nursing home, but energy/weight per serving varied depending on the meal served and condition).Variety: Participants served main meal with 1 sauce: white sauce, gravy, chasseur (vegetable-based) sauce, or mustard sauce.Control: Participants served main meal (same as main meal presented in the variety condition), without sauce.Outcome: Grams (weighed). Energy consumed (kJ) also reported. Intake was averaged across the 2 test meals for each condition	Significant effect of variety, but only when energy intake is reported; participants consumed more food in the variety condition compared with control	Energy consumed from protein and fat components was significantly higher in meals with sauce compared with no sauce. No significant differences in energy consumed from carbohydrate or vegetable components were found. No significant differences were found in weight consumed for any individual components	No significant variety × “expectancy of effect” interaction found for meal intake	No sample size calculation; control procedures included a washout period between conditions and presenting the test meal at the standard mealtime in the nursing home; baseline assessment of hunger and desire to eat before each meal reported; pleasantness and familiarity assessed after meal; no cover story was used; participants were tested in a communal dining hall (social setting is a potential confound); participant expectations of effects of sauce (would affect intake compared with do not know) were included in model, and no significant differences were found between conditions for hunger, desire to eat, pleasantness, familiarity, and energy provided
Appleton (2018), UK^3^ (33)	Laboratory; within-subjects; randomized order of conditions, but method is unclear.Sample = 56 older adults (31 females).Age = 71.1 y, SD = 4.6.BMI = 25.8 kg/m^2^, SD = 2.5.Exclusions *N* = 4 (*N* = 2 did not return for second visit, *N* = 2 self-reported failure to follow experimental procedures)	Variety manipulated within a single course; sauces were provided with a lunch meal consisting of chicken (300 g), sweetcorn (250 g), carrots (250 g), and mashed potatoes (325 g) (whole-meal serving excluding sauce = 3900 kJ). In both conditions, participants were also served an evening meal 4.5 h after lunch on each test day that was not manipulated for variety (consisting of a selection of cold buffet meal foods and condiments, serving = 17, 890 kJ).Variety: Participants served lunch with 1 sauce: chicken gravy (212 kJ).Control: Participants served lunch (same as main meal presented in the variety condition), without sauce.Outcome: Grams (weighed). Energy consumed (kJ) also reported	Significant effect of variety for both energy and weight consumed; participants consumed more food in the variety condition compared with control	No significant effect of variety for energy or weight consumed when intake from sauce in the variety condition was excluded from analyses. Weight consumed from protein, fat, and carbohydrate was significantly lower in the control condition compared with the variety condition (including intake from sauce). When intake from sauce was excluded from analyses, a significant difference was found for protein only; weight consumed from protein was significantly lower in the control condition compared with the variety condition	Significant variety × protein intake interaction (participants grouped as increased protein intake compared with decreased protein intake in response to sauce); participants who had increased protein intake had significant increases in energy, protein, and fat in the variety condition compared with control (when intake from sauce was excluded from analyses) and significant increases in weight, energy, protein, fat, and carbohydrate in the variety condition compared with control (when intake from sauce was included in analyses). Participants who had decreased protein intake had significant decreases in energy, protein, fat, and weight in the variety condition compared with control (when intake from sauce was excluded from analyses) and significant decreases in energy and protein and significant increases in weight in the variety condition compared with control (when intake from sauce was included in analyses)	No sample size calculation; control procedures included a washout period between conditions, presenting the test meal at a standard mealtime, instructions to consume the same breakfast on each test day and abstain from eating/drinking between breakfast and lunch (excluding water), instructions to refrain from drinking alcohol or doing heavy exercise 1 d prior to or on the day of the test session, and participants self-reported compliance with control procedures; pre-post assessment of hunger, desire to eat, thirst, and desire to drink for each meal reported; pleasantness, tastiness, and familiarity assessed after each meal; no information about cover story; no additional confounds identified; no significant differences were found between conditions for location, but significant differences in pleasantness, tastiness, familiarity, and desire to eat were reported (variables were not included in model for main effect)
Beatty (1982), US^4^ (21)	Laboratory; between-subjects; random assignment to conditions, but no method given.Sample = 22 students (12 females).Age = not reported.BMI = not reported. No exclusions	Variety manipulated within a single course; test meal included ice cream only (portion self-selected by participant).Variety: Three flavors of ice cream that participants liked the most out of 4 possible choices, served in a single mixed bowl.Control: One flavor of ice cream that participants liked the most, served in a single bowl.Outcome: Grams (weighed)	Significant effect of variety for females but not males; females in the variety condition consumed more ice cream than females in the control condition	None reported	None reported	No sample size calculation reported; control procedures included instructions to eat a normal dinner and skip dessert the evening before the study; no assessment of appetite reported; liking and frequency of consumption assessed before study; no cover story was used; food intake was assessed in groups (social setting is a potential confound), and portions self-selected (potential confound); no additional variables controlled in models
Bergamaschi (2016), Denmark^5^ (34)	Field (school); within-subjects; randomized order of conditions, but no method given.Sample = 153 children (63 females).Age = 9.6 y, SEM = 0.05.BMI = 17.2 kg/m^2^, SEM = 0.2.Exclusions *N* = 18 (included in pilot study only)	Variety manipulated within a single course; test meal included fruit and vegetables only (serving = ∼200 g). Two sets of stimuli were served; “classical variety” varied the number of different test foods presented, and “perceived variety” varied the shape of test foods only.High variety, classical: Carrot, green apple, plum, white cabbage, dried cranberry, almond.High variety, perceived: Carrot (chunks, slices, sticks), green apple (chunks, slices, triangles).Medium variety, classical: Carrot, green apple, white cabbage, dried cranberry.Medium variety, perceived: Carrot (chunks, sticks), green apple (chunks, slices).Control, classical: Carrot, green apple.Control, perceived: carrot (chunks), green apple (chunks).Outcome: Grams and kcal (weighed), expressed as percent	Significant effect of variety only when the classical variety set was presented; intake was higher in the control condition compared with the high- and medium-variety conditions, respectively. There were no significant differences in intake between conditions when presented with the perceived variety set	None reported	None reported	No sample size calculation reported; control procedures included a washout period between conditions, instructions to parents to not give snacks to children before the study, and food intake measured at usual snack time in usual setting; no assessment of appetite reported; food liking, familiarity, and frequency of consumption were assessed before and after the main study sessions; no information about cover story; food intake was assessed in groups (social setting is a potential confound) and significant differences in liking between test foods (potential confound, does not appear to have been controlled for in models); “children” and “class” were considered random factors; age and BMI *z* score were considered covariates in models
Best (2011), UK (35)	Laboratory; within-subjects; counterbalanced order of conditions, but method is unclear.Sample = 18 older adults (14 females).Age = 77.0 y.BMI = 30.0 kg/m^2^.No exclusions	Variety manipulated within a single course; choice seasonings and sauces were provided for a main meal consisting of chicken, 2 types of vegetables, and mashed potatoes (portion sizes for each food item were double the “usual” portion for adults, 2 teaspoons of seasoning were added to chicken, and ∼100 g of sauce was added to chicken).Variety, seasoning: Participants given a choice of 1 seasoning to be added to meal: chargrilled chicken seasoning, Cajun seasoning, smoky bacon seasoning, lemon and herb peri-peri seasoning rub, lime and coriander peri-peri marinade, or sundried tomato and basil peri-peri marinade.Variety, sauce: Participants given a choice of 1 sauce to be added to meal: chicken gravy, onion gravy, honey and mustard sauce, creamy mushroom sauce, creamy stroganoff sauce, or tomato and basil sauce.Control: Participants served meal with no sauce or seasoning.Outcome: Grams (weighed). Energy consumed (kcal) also reported	Significant effect of variety for both energy and weight consumed; participants consumed more energy in the seasoning and sauce variety conditions compared with control, and participants consumed more weight of food in the sauce variety condition compared with the seasoning variety and control conditions, respectively. No significant differences in energy intake between seasoning and sauce variety conditions, and no significant differences in weight of food consumed between the seasoning variety and control conditions	Intake (in grams) from protein and carbohydrate was significantly greater in the seasoning and sauce variety conditions compared with control, total fat intake was significantly greater in the sauce variety condition compared with the seasoning variety and control conditions, and total fat intake was significantly greater in the seasoning variety compared with control conditions. Weight of chicken consumed was significantly greater in the seasoning and sauce variety conditions compared with control. All other comparisons were not significant for energy/weight consumed from individual food components. When intake from seasonings and sauces was excluded from analyses, no significant differences were found between conditions for carbohydrate intake or weight of food consumed, and between sauce and seasoning variety conditions for fat intake	None reported	No sample size calculation; control procedures included a washout period between conditions, presenting the test meal at a standard mealtime, and instructions to consume the same breakfast at the same time on each test day; pre-post assessment of hunger and desire to eat reported; pleasantness and familiarity assessed after each meal; no information about cover story; food intake was assessed in groups (social setting is a potential confound, although participants were seated in individual booths and asked not to communicate with other participants); no significant differences were found between conditions for hunger, desire to eat, or familiarity, but significant differences in pleasantness and flavor intensity were reported (variables were not included in model for main effect)
Carney (2018), US (36)	Laboratory; within-subjects; counterbalanced order of conditions, but no method given.Sample = 44 children (19 females).Age = 54.2 mo, SD = 8.2.BMI *z* score = 0.2, SD = 1.0.Exclusions *N* = 35 (*N* = 15 did not meet eligibility criteria, *N* = 16 had scheduling conflicts, *N* = 4 refused participation and/or would not eat the test foods)	Variety manipulated within a single course; test meal included macaroni and cheese (175 g), unsweetened applesauce (115 g), carrots (3 × 40-g servings), and 1 energy-containing beverage (milk).Variety: Carrots seasoned with 3 different spice blends (cinnamon, nutmeg, and ginger; cardamom, cumin, and allspice; and garlic, black pepper, and oregano). Each flavor was presented in a separate bowl and served as side dishes at the meal.Control: Carrots were all served with the cinnamon spice blend, but participants were again presented with 3 servings in separate bowls to be consistent with the variety condition.Outcome: Grams (weighed). Energy and proportion consumed also reported	No significant effect of variety on food intake	No significant effect of variety on the amount of individual meal items consumed (macaroni and cheese, apple sauce, carrots). Also, no significant effect of variety on beverages consumed (milk, water)	Significant propylthiouracil (PROP) status × variety interaction reported for carrots consumed; children categorized as PROP tasters consumed more carrots in the variety condition compared with PROP nontasters, and PROP nontasters consumed more carrots in the control condition compared with tasters but only when intake was expressed as a proportion. No significant differences in intake between conditions within groups of tasters or nontasters	No sample size calculation reported; control procedures included a washout period between conditions and meals given at usual time for lunch or dinner; pre-post assessment of fullness and liking reported, and parents completed questionnaires on their child's usual eating habits; no information about cover story; no additional confounds identified; age, sex, BMI *z* score, meal order, mealtime, and selection of the cinnamon carrot as the favorite were considered potential covariates but removed from final models if not significant
Carstairs (2018), UK (37)	Field (children's nursery); within-subjects; counterbalanced order of conditions using Latin squares for each nursery group and by alternating first portion size block. Sample = 43 children (23 females).Age = 3.9 y, SEM = 0.6.BMI = 16.5 kg/m^2^, SEM = 1.3.Exclusions *N* = 15 (*N* = 7 did not attend lunch sessions, *N* = 2 withdrew from the study, *N* = 6 ate <10% of meal)	Variety manipulated within a single course; test meal included a cheese sandwich (117 g or 70 g, depending on serving size condition) and vegetables (120 g). Grapes (40 g) and yogurt (120 g) were served after the test meal.Variety: Vegetables include equal servings of cucumber, tomato, and carrot to accompany cheese sandwich.Control: Cucumber, tomato, or carrot served to accompany cheese sandwich.Outcome: Grams (weighed). Energy consumed (kcal) also reported. Mean single vegetable intake (collapsed across 3 sessions) reported for control condition	No significant effect of variety on food intake	Significant effect of variety on vegetable intake; participants consumed more vegetables in the variety condition compared with the control condition. No significant effect of variety on sandwich intake	No significant portion size (large or small sandwich) × variety interaction effect found for total meal, sandwich, or vegetable intake	Target sample *N* = 48 reported (effect size = 0.5, 1 − β = 0.80, α = 0.05); control procedures included a washout period between conditions and meals given at the usual time for lunch in normal setting; no assessment of appetite reported; baseline rating of liking assessed, and parents completed questionnaires on their child's usual eating habits; no information about cover story; food intake was assessed in groups (social setting is a potential confound, although food sharing/dropped items were monitored); no additional variables controlled in main model, regression analysis was used to identify additional predictors of HED and LED intakes (mean intakes, age, BMI, eating traits, and parental feeding practices)
Divert (2015), France^6,7^ (38)	Field (nursing home); within-subjects; counterbalanced order of conditions using a method of alternation. Sample = 42 older adults (29 females). Age = 86.6 y.BMI = Not reported. Exclusions *N* = 12 (did not complete all conditions)	Variety manipulated within a single course; test meal menu composed of a starter, a main course, a dairy product, and a dessert. Variety was manipulated for the main course vegetable accompaniment (serving = 150 g) and condiments (freely available throughout meal).Variety, vegetables: Served green beans and zucchinis.Control, vegetables: Served green beans only.High variety, condiments: Offered salt, pepper, mustard, butter, vinaigrette, tomato sauce, mayonnaise, garlic, shallot, lemon, parsley (11 condiments).	Significant effect of vegetable variety on food intake; total meal intake was greater when participants were served vegetables in the variety condition compared with the control condition. No significant effect of condiment variety on total meal intake	A significant effect of condiment variety was found on garnish intake (rice), but no effect was found for meat, dairy product, or dessert; participants ate more rice when offered 7 or 11 condiments compared with 3 condiments. A significant effect of vegetable variety was also found for meat intake; intake was greater when participants were given 2 vegetables compared with when they were given a single vegetable	None reported	No sample size calculation reported; control procedures included a washout period between conditions and meals given at the usual time for lunch in usual setting; baseline assessment of hunger reported; meal enjoyment assessed after eating; no information about cover story; food intake appears to be assessed in groups (social setting is a potential confound), alternative food products were given to participants with chewing/bowel issues (influence on satiation is a potential confound), and piece count increases risk of researcher bias; no additional variables controlled in models
		Medium variety, condiments: Offered salt, pepper, mustard, butter, vinaigrette, tomato sauce, mayonnaise (7 condiments).Control, condiments: Offered salt, pepper, mustard (3 condiments).Outcome: Grams (weighed), reported as energy consumed (kcal). Piece count used to measure condiment intake				
Domínguez (2013), Spain (39)	Field (school); between-subjects; method of assignment to conditions is unclear. Sample = 152 children (sex not reported).Age range = 4–6 y.BMI = Not reported. Exclusions *N* = 61 (*N* = 2 were vegetarians, reason for exclusion unclear for *N* = 59)	Variety manipulated within a single course; test meal included vegetables only (serving = 149 g).Variety: Served both green beans and zucchini.Control, choice: Given a choice of either green beans or zucchini.Control, no choice: Served either green beans or zucchini.Outcome: Grams (weighed)	Significant effect of variety on food intake; children ate significantly more vegetables in the variety condition compared with control (no choice), and they also ate significantly more vegetables when given a choice compared with no choice in the control condition	None reported	Interactions explored with school, age, and sex; no significant interaction effects were found	No sample size calculation reported; control procedures included meals given in the usual setting for lunch in school environment; no assessment of appetite reported; baseline assessment of liking reported, and this was used to select test foods for the main study; no information about cover story, although efforts were made to conceal condition allocation from children (e.g., children in the same class were assigned to the same condition); food intake assessed in groups (social setting is a potential confound, as parents and teachers were present); school, age, and sex were controlled in models
Epstein (2013), US [study 1]^8^ (40)	Laboratory; between-subjects; random assignment to conditions stratified by sex, but no method given. Sample = 31 children (14 females).Age = 10.4 y, SD = 1.3.BMI *z* score = 1.8, SD = 0.5. Exclusions *N* = 3 (did not follow experimental procedures)	Variety manipulated across meals; baseline test meal of Kraft macaroni and cheese was served on days 1 and 5 across conditions, and high energy-dense foods were served on days 2 to 4 (meal serving = 1200 kcal).High variety: Served 1 of 3 of their highest-rated energy-dense foods on each test day (foods rated at baseline; chicken nuggets, 4-cheese pizza, mozzarella sticks, cheeseburgers, fish sticks). Foods were served with condiments (30-kcal portions of honey mustard, pizza sauce, barbeque sauce, and tomato ketchup).Medium variety: Served 1 of 3 of their highest-rated macaroni and cheese dishes on each test day (foods rated at baseline; Kraft Spiral, SpongeBob SquarePants, Cheddar Explosion, Whole Grain and White Cheddar Macaroni and Cheese).Control: Served Kraft Macaroni and cheese on each test day.Outcome: Grams (weighed), reported as energy consumed (kcal)	Across days 2–4, there was a significant effect of variety on food intake; children consumed significantly more energy each day in the high-variety condition compared with the medium-variety and control conditions. There were no significant differences between the medium-variety and control groups	For days 2–4, the effect of variety was unchanged when calories from condiments were excluded from analyses	Significant variety × session interaction for days 1 and 5; children in the control and medium-variety groups reduced their intake from sessions 1 to 5, where children in the variety group increased their intake from sessions 1 to 5. There were no differences in intake between the medium-variety and control groups	No sample size calculation reported; control procedures included instructions to eat a normal breakfast and lunch on each test day, instructions to abstain from eating and drinking for 3 h prior to the test session (excluding water), abstain from eating test foods for 24 h prior to test day, abstain from eating macaroni and cheese for whole study period (dietary recall questionnaires were completed to check compliance), and testing during usual dinner time; pre-post assessments of hunger reported for each test session; liking of test foods assessed on days 1 and 5; no information about cover story; no additional confounds identified; baseline hunger ratings controlled in models
Guerrieri (2007), Netherlands (19)	Laboratory; between-subjects; random assignment to conditions, but no method given.Sample = 86 students (females only).Age = 20.2 y, dispersion = 3.4 (measure of variability unclear).BMI = 21.8 kg/m^2^, dispersion = 3.0 (measure of variability unclear).No exclusions	Variety manipulated within a single course; test meal included candies only (serving = 1600 g).Variety: Served 14 different colors of sugar beans. Control: Served plain white sugar beans only.Outcome: Grams (weighed)	No significant effect of variety on food intake	None reported	No significant impulsivity × variety interaction effect found when participants were categorized as high or low impulsive, for food intake	No sample size calculation; control procedures included instructions to eat a small meal/snack 2 h before the test session and to otherwise abstain from eating and drinking prior to test session (excluding water); no indication ofmeasurement of appetite on the test day, but participants self-reported compliance with control procedures; no assessment of food liking; cover story was used but no indication as to whether this was believed by participants; no additional confounds identified; no additional variables controlled in main model
Guerrieri (2008), Netherlands^9^ (41)	Field (school); between-subjects; random assignment to conditions via coin toss.Sample = 78 children (33 females).Age = 9.0 y, dispersion = 0.6 (measure of variability unclear).BMI = 17.4 kg/m^2^, dispersion = 2.6 (measure of variability unclear).No exclusions	Variety manipulated within a single course; test meal included candies only (serving = 350 g).Variety: Five sorts of marshmallows served in a single bowl: white-pink marshmallows, pink marshmallows covered in coconut, white marshmallows covered in coconut, marshmallows covered in milk chocolate, and yellow and green marshmallows in different forms.Control: White-pink marshmallows only, served in a single bowl.Outcome: Grams (weighed), reported as energy consumed (kcal)	No significant effect of variety on food intake	None reported	There was a significant reward sensitivity × variety interaction effect found; children in the variety condition who were more reward sensitive consumed greater energy than the less reward-sensitive children. There was no difference in food intake between more and less reward-sensitive children in the control group. There was no significant response inhibition × variety interaction effect or 3-way interaction effect (response inhibition × reward sensitivity × variety)	No sample size calculation; children asked to self-report intake on the test day and a day before, and children were tested between school break times; baseline hunger assessed; liking assessed during Bogus Taste Test, but these data do not appear to have been included in data analyses; cover story was used, but no indication as to whether this was believed by participants; no additional confounds identified; age controlled in main model as a significant covariate (hunger, BMI, and sex were also assessed as potential covariates)
Guerrieri (2012), Netherlands (42)	Laboratory; between-subjects; random assignment to conditions, but no method given.Sample = 80 students (females only).Age = not reported.BMI = 22.8 kg/m^2^, dispersion = 3.4 (measure of variability unclear).Exclusions *N* = 3 (did not indicate hunger levels)	Variety manipulated within a single course; test meal consisted of an assortment of cookies (serving = 400 g).Variety: Chocolate chip cookies, coconut macaroons, sponge-like biscuits filled with orange jam and covered in bitter chocolate, and milk chocolate–covered cookies filled with vanilla cream.Control: Chocolate chip cookies only.Outcome: Grams (weighed), reported as energy consumed (kcal)	Variety was a significant predictor of food intake (no descriptive statistics reported)	None reported	The interactions reward × variety and hunger × variety were not significant. However, there was a significant 3-way interaction (hunger × reward × condition), as participants would consume more food when hungry if they were more reward sensitive but only in the variety condition	No sample size calculation; control procedures included instructions to eat a small meal/snack 3 h before test session, to otherwise abstain from eating and drinking prior to test session (excluding water), and participants self-reported compliance with control procedures; baseline assessment of hunger; no assessment of food liking; cover story was used, and participant awareness of aims/conditions was checked; no additional confounds identified; no additional variables controlled in main model
Hollis (2007), UK (20)^10^	Laboratory; within-subjects; counterbalanced order of conditions, but no method given. Sample = 36 young and older adults (18 females) (participants grouped by age).Young adults age = 25.0 y, SD = 4.0.Older adults age = 72.0 y, SD = 6.0.Young adults’ BMI = 22.7 kg/m^2^, SD = 2.6.Older adults’ BMI = 26.5 kg/m^2^, SD = 3.8.No exclusions	Variety manipulated across courses; test meal included sandwiches only (serving size unknown).Variety: Sandwiches with a different filling in each of 4 courses: grated cheese, cream cheese and cucumber, ham, turkey.Control: Sandwiches with cheese filling for each of 4 courses.Outcome: Grams (weighed)	Significant effect of variety on food intake; both young and older adults consumed more sandwiches in the variety condition compared with control	None reported	Significant age × variety interaction; older adults consumed more sandwiches in the control condition compared with young adults, and young adults consumed more sandwiches in the variety condition compared with older adults.Significant course × variety interaction; food intake at each course declined more during the control condition compared with the variety condition	No sample size calculation; control procedures included screening for eating disorders, restraint, and depression, a washout period between conditions, and instructions to eat usual breakfast and fast for 3 h before session on test day; baseline assessment of hunger, thirst, and fullness reported; liking assessed during study screening; cover story was used, but no indication as to whether this was believed by participants; no additional confounds identified; no additional variables controlled in main model (no significant differences in hunger, thirst, or desire to eat were identified)
Kerr (2019), Australia (43)	Laboratory; between-subjects; random assignment to conditions according to test day by independent statistician.Sample = 1274 parents (86% female) and 1299 children (50% female).Adult age = 43.9 y, SD = 5.1.Children age = 11.4 y, SD = 0.5.Adult BMI = 27.8 kg/m^2^, SD = 6.1.Children BMI *z* score = 0.3, SD = 1.0.Exclusions *N* = 41 children, 60 adults (food allergies)	Variety manipulated within a single course; test meal consisted of snack assortment (serving for children = 181 g control or 240 g variety) (serving for adults = 207 g control or 266 g variety).Variety: Children received a small or larger box containing 8 snacks: peaches or fruit salad, flavored rice crackers, miniature wheat fruit bites, cheese wedge, mini animal-shaped biscuits, fruit muesli/granola bar 1, miniature milk chocolate bar 1, miniature milk chocolate bar 2. Adults received the same items excluding mini animal-shaped biscuits and additionally received mini Oreo biscuits and fruit muesli/granola bar 2 (for a total of 9 items).Control: Children received a small or larger box containing 5 snacks: peaches or fruit salad, flavored rice crackers, cheese wedge, mini animal-shaped biscuits, miniature milk chocolate bar 1. Adults received the same items excluding mini animal-shaped biscuits and additionally received mini Oreo biscuits and fruit muesli/granola bar 1 (for a total of 6 items).Outcome: Grams (weighed). Energy consumed (kJ) also reported	Significant effect of variety for children; children consumed more food in the variety condition compared with control. Significant effect of variety for adults, but only when intake was measured in kJ; adults consumed more energy in the variety condition compared with control	None reported	No significant interaction for the box size × variety for children, but a significant interaction for adults. In the control condition, adults consumed less food when presented with a large rather than small box. In the variety condition, adults consumed more food when presented with a large rather than small box	No sample size calculation; control procedures included a semifasting venipuncture 5–30 min before the test session; baseline assessment of hunger reported; no assessment of liking; cover story was used, and participant awareness of aims/conditions was recorded; food intake appears assessed in groups, although participants were seated separately (social setting is a potential confound), and participants were able to complete other activities while eating (e.g., reading, look at phone); no additional variables controlled in main model, but sensitivity analyses adjusted for age, sex, socioeconomic status, BMI, puberty status, baseline hunger, fasting times, and duration spent in the session. Separate analyses also removed participants who expressed awareness about being monitored, had >10% of food wrappers missing from their box after eating, had fruit salad instead of peaches, or had 11-g rather than 13-g chocolate bars
Levitsky (2012), US [study 1]^11^ (44)	Laboratory; within-subjects; randomized order of conditions, but no method given. Sample = 27 students (4 females).Age range = 18–21 y.BMI = Not reported. Exclusions *N* = 8 (did not complete the study)	Variety manipulated within a single course; 1 of 2 meals with a protein (91 g in meal 1 and 78 g in meal 2), carbohydrate (86 g in meal 1 and ½ cup in meal 2), and vegetable component (81 g in meal 1 and 89 g in meal 2), served in separate bowls, was presented.Variety, composite: Participants served protein, carbohydrate, and vegetable in each meal; chicken tenders, potato tots, and green beans in meal 1; chicken fillets, rice, and peas in meal 2.Control, low carb: Participants served protein and vegetable component in each meal; chicken tenders and green beans in meal 1; chicken filets and peas in meal 2.Control, vegetarian: Participants served carbohydrate and vegetable component in each meal; potato tots and green beans in meal 1; rice and peas in meal 2.Outcome: Grams (weighed). Energy consumed (kcal) also reported. Intake was averaged across the 2 test meals for each condition	Significant effect of variety for intake in both energy and grams; total food intake averaged across meals was greater in the variety condition (3 components in a composite meal) compared with when participants were served the low-carb or vegetarian meals (2 components in a meal)	Energy and gram intake varied across conditions for individual components; protein and vegetable intake were significantly greater in the low-carb meal compared with the composite meal, and carbohydrate and vegetable intake was significantly greater in the vegetarian meal compared with the composite meal	No significant interaction for test meal (1 or 2) × individual components (protein, vegetable, or carbohydrate) on food intake	No sample size calculation; control procedures included a washout period between conditions, instructions to eat the same food in the same amount prior to the session on each test day, and instructions to maintain the same level of physical activity on each test day; no assessment of appetite reported; no assessment of liking reported; cover story was used, but referred to caloric intake as a measure of interest; food intake was assessed in groups (social setting is a potential confound); no additional variables included in models
Meengs (2012), US (17)	Laboratory; within-subjects; counterbalanced order of conditions using a Latin square. Sample = 69 adults (34 females).Male age = 27.4 y, SEM = 1.2.Female age = 25.5 y, SEM = 0.6.Male BMI = 25.5 kg/m^2^, SEM = 0.6.Female BMI = 23.3, SEM = 0.6.^12^Exclusions *N* = 3 (“highly variable” food intake)	Variety manipulated within a single course; test meal included pasta with sauce (600 g) and cooked vegetables (600 g) on a single plate.Variety: Served 3 vegetables to accompany pasta: broccoli, baby carrots, and snap peas.Control: Served 1 of 3 vegetables to accompany pasta: broccoli, baby carrots, or snap peas.Outcome: Grams (weighed). Energy consumed (kcal) also reported	Significant effect of variety on total meal intake for both men and women; participants consumed more in the variety condition compared with control, but only when intake was reported in grams. Total meal energy intake was only significantly different for men, but this was unrelated to vegetable intake	Significant effect of variety on vegetable intake for both men and women; participants consumed more in the variety condition compared with control (for all 3 individual vegetables and when compared with most preferred vegetable). There was no significant effect of variety on pasta intake (in grams or kcal)	There was a significant sex × variety interaction, as pasta intake differed across conditions for men; they consumed more pasta and greater energy overall in the variety and peas-only conditions compared with carrots only and broccoli only in the control. However, this was unrelated to vegetable intake	No sample size calculation; control procedures included a washout period between conditions, a standard breakfast at least 3 h before the test session, instructions to abstain from eating/drinking between breakfast and test session (excluding water), and participants self-reported compliance with control procedures; pre-post assessment of hunger, fullness, nausea, and prospective consumption reported; baseline assessment of pleasantness reported, and participants ranked vegetables for preference at the end of the final test session; no information about cover story; no additional confounds identified; “participant” was treated as a random effect, and sex and study week were included as fixed effects in main model
Meiselman (2000), US (4)	Laboratory; between-subjects; no information about allocation to conditions.Sample = 47 adults (9 females).Variety group age = 44.8 y.Control group age = 44.8 y.Variety group weight = 82.5 kg and height = 174.7 cm.Control group weight = 85.2 kg and height = 177.4 cm.No exclusions	Variety manipulated across meals; baseline meal of Swedish-style meatballs (150 g), mashed potatoes (215 g), green beans (120 g), and gravy (55 g) served on days 1 and 5 across conditions, and meals containing a main dish of meat/fish, vegetable, and starch served on days 2–4 (50% above package serving recommendation for each meal).Variety: Day 2 meal composed of veal parmigiana with tomato sauce, potato wedges, and mixed vegetables; day 3 meal composed of crunchy fish fillets, deep fries, and broccoli; day 4 meal composed of chicken nuggets, macaroni and cheese, broccoli, cauliflower, and carrots.Control: Served baseline meal of Swedish-style meatballs, mashed potatoes, green beans, and gravy on days 2–4.Outcome: Grams (weighed)	None reported	Significant pre-post change in intake for the baseline meal in both conditions. In the variety group, intake significantly increased for meatballs, green beans, and mashed potatoes from day 1 to day 5. In the control group, intake significantly decreased for meatballs and green beans from day 1 to day 5	None reported	No sample size calculation; no control procedures; no assessment of appetite; liking/acceptance measured for each food and each overall meal on each day; no cover story, as participants responded to an advert for a free meal from the “Food Acceptance Laboratory,” but it is mentioned that participants were unaware of food weighing; food intake assessed in groups (social setting is a potential confound); no additional variables controlled in main model
Mok (2010), Singapore (45)	Laboratory; within-subjects; counterbalanced order of conditions using Latin square. Sample = 63 students (females only).Age range = 18–20 y. BMI range = 17.4–26.5 kg/m^2^. Exclusions *N* = 39 (*N* = 21 did not sign up for main study after screening, *N* = 16 did not meet study criteria, *N* = 2 declined participation)	Variety manipulated across courses; test meal consisted of chocolates only (serving = 200 g).Variety: Participants given a serving of a different chocolate for each of 3 courses: M&Ms, Kinder Bueno, or Swiss dark Toblerone.Control: Participants given a serving of their favorite chocolate in all 3 courses: M&Ms, Kinder Bueno, or Swiss dark Toblerone (determined during study screening for liking of test foods).Outcome: Grams (weighed), converted to kilojoules consumed	No significant effect of variety	None reported	There was a significant restraint × variety interaction; participants categorized as “unrestrainers” (*N* = 12) ate significantly more chocolate in the variety condition than in the plain condition.There was no significant interaction reported for course × variety	No sample size calculation; control procedures included a washout period between conditions, meals given at usual time for lunch or dinner, instructions to abstain from all foods and drinks for at least 2 h before the test session, instructions to arrive on each test day in approximately the same state of hunger, and participants self-reported their intake at lunch before the first test session; baseline assessment of hunger reported; acceptance of test foods was measured before the main study, and pre-post meal assessment of pleasantness reported; cover story was used, but no indication as to whether this was believed by participants; eating behavior questionnaire completed before intake was measured (potential confound); no additional variables controlled in main model
Norton (2006), UK (46)	Laboratory; within-subjects; counterbalanced order of conditions using Latin square.*N* = 30 adults (15 females).Age = 29.5 y, SD = 12.6.BMI = 23.6 kg/m^2^, SD = 3.1.Exclusions *N* = 1 (outlier in data analyses)	Variety manipulated within a single course; test meal included sandwiches with different fillings, served after either a low- or high-volume soup preload (serving = 2216 kcal for men and 1662 kcal for women).Variety: Participants served sandwich quarters with their second, third, and fourth preferred fillings: cheese, salami, tuna, or chicken.Control: Participants served sandwich quarters with their second preferred filling: cheese, salami, tuna, or chicken.Outcome: Grams (weighed). Energy consumed (kcal/kJ) also reported	Significant effect of variety for intake in both energy and grams; total food intake was greater in the variety condition compared with the control condition	Significant effect of preference within the variety condition; intake of the fourth preferred filling was significantly lower than the second and third preferred fillings. However, in the variety condition, the tendency to consume all 3 fillings was significant	No significant interaction for soup preload × variety, sex × variety, DEBQ × variety, TFEQ × variety, or sex × variety × preload on food intake	Target sample *N* = 20 reported (effect exceeding 15% reduction in intake by preload volume, 1 − β = 0.80, α = 0.05); control procedures included a washout period between conditions, instructions to fast from midnight the night before each test day until arrival at the laboratory, a standardized breakfast served on the morning of each test day, instructions to abstain from eating and drinking between breakfast and lunch (excluding 1.5 L water), and compliance with procedures was checked; pre-post assessment of hunger, fullness, desire to eat, and prospective consumption reported (following soup preload and sandwich lunch); baseline assessment of liking before main study reported; cover story was used, but no awareness check was reported; no additional confounds identified; BMI was included as a between-subjects factor
Parizel (2017), France (47)	Laboratory; within-subjects; counterbalanced order of conditions using Williams Latin square.Sample = 59 adults (47 females).Age = 27.4 y, SD = 6.8.BMI = 21.7 kg/m^2^, SD = 2.1.Exclusions *N* = 5 (*N* = 2 did not attend all sessions, *N* = 3 underestimated their weight and had a BMI >26 kg/m^2^)	Variety manipulated within a single course; test meal included a main course of ham (served amount that they had consumed ad libitum in the first session) and vegetables (serving = 400 g). After the test meal, participants were served apple puree (350 g) and a cup of flavored tea.Variety, no choice: Served 3 vegetable dishes side by side during the main course: spinach, green beans, and zucchini.Variety, choice: Presented with 3 vegetable dishes during the main course, and participants could choose to have 1, 2, or all 3 vegetables.Control, no choice: Served 1 vegetable dish during the main course, randomly selected from spinach, green beans, and zucchini.Control, choice: Presented with 3 vegetable dishes, and participants could choose to have 1 vegetable only.Outcome: Grams (weighed). Energy (kcal) consumed also reported for total meal intake	No significant effect of variety	None reported	There was no significant interaction reported for choice × variety	No sample size calculation; control procedures included a washout period between conditions, instructions to have the same breakfast on each test day, a standardized mealtime across participants, and instructions to abstain from eating or drinking for at least 3 h before the test session (except water); assessment of hunger before eating, after the main course, and after each total meal reported; liking for each vegetable dish assessed after the meal and at the end of the last session; cover story was used, but no indication as to whether this was believed by participants; food intake assessed in groups (social setting is a potential confound, although participants were seated in individual booths to reduce influence); “participant” included as a random factor, and order of sessions was included as a fixed factor in models; sensitivity analyses adjusted for plate clearers
Pliner (1980), Canada^13^ (48)	Laboratory; between-subjects; no information about allocation to conditions.Sample = 103 students (males only) (participants grouped by weight and dieting status).Participants in “obese weight” group age = 19.5 y.Participants in “lean weight” group age = 19.8 y.Participants in “obese weight” group weight = 197.9 lbs and height = 69.5 in.Participants in “lean weight” group weight = 152.7 lbs and height = 69.5 in.Exclusions *N* = 31 (*N* = 12 did not meet criteria for weight classifications for the study, *N* = 6 had median scores on the dieting scale, *N* = 8 consumed only 1 type of hors d'oeuvre in the variety condition, *N* = 5 were randomly excluded for equal groups)	Variety manipulated within a single course; test meal consisted of an assortment of hors d'oeuvres (serving = 30 pieces).Variety: Served 3 types of hors d'oeuvres: sausage rolls, pork and shrimp egg rolls, and pizza slices.Control: Served 1 type of hors d'oeuvre: sausage rolls, pork and shrimp egg rolls, or pizza slices.Outcome: Piece count (number of hors d'oeuvres eaten)	Significant effect of variety; across weight classification groups, participants ate more hors d'oeuvres in the variety condition compared with control	None reported	There was no significant interaction reported for weight classification × variety	No sample size calculation; control procedures included instructions to abstain from eating for at least 4 h before the test session; pre-post assessment of hunger and time of last eating reported; liking assessed for food assortment after the test meal; cover story was used, but no indication as to whether this was believed by participants; food intake assessed using piece count (a potential confound); no additional variables controlled in main model (although it is reported that there were no significant differences in hunger or time of last eating between conditions)
Raynor (2006), US (22)	Laboratory, with at-home participation; between-subjects; random allocation to conditions, but no method given. Sample = 29 students (males only).Control group age = 20.2 y, SD = 2.0.Variety group age = 20.7 y, SD = 2.5.Control group BMI = 23.3 kg/m^2^, SD = 1.9.Variety group BMI: 22.9 kg/m^2^, SD = 2.4.Exclusions *N* = 8 (*N* = 4 indicated liking <50 for one of the test foods; *N* = 2 had a BMI >30, *N* = 1 did not attend first session; *N* = 1 did not follow study instructions)	Variety manipulated across meals; baseline snack of crumb cake (serving = 375 g) to eat in the laboratory on days 1 and 4 across conditions, and snacks were served on days 2–4 in the laboratory and taken home on days 1–3 (serving = 375 g for each snack).Variety: Day 1 take home peanuts, day 2 eat Oreo cookies in the laboratory and take home tortilla chips, day 3 eat potato chips in the laboratory and take home chocolate chip cookies.Control: Take home crumb cake on days 1–3, and eat crumb cake in the laboratory on days 2 and 3.Outcome: Grams (weighed), converted to kcal consumed	There was no significant effect of variety condition on energy intake from crumb cake on days 1 and 4. Total intake for each snack on days 2–4 was not reported. Total intake across days was not reported	None reported	There was no significant interaction reported for time (day) × variety	No sample size calculation; control procedures included instructions to eat in their usual eating pattern before each session, to abstain from eating for 3 h before each session, and participants self-reported intake prior to the session to check compliance; pre-post assessment of hunger in each session reported; liking assessed for each food on days 1 and 4; no information about cover story; food intake assessed in cafeteria (social setting is a potential confound, although participants were seated at individual tables facing away from other participants to reduce influence); no additional variables controlled in main model (no significant differences found for any baseline characteristics between conditions; no significant differences in liking for foods between conditions on day 1; no significant differences between conditions and days for hunger, hours since last eaten, or intake prior to test session)
Raynor (2012), US (18)	Laboratory; within-subjects; random order of conditions stratified by sex, but no method given. Sample = 24 adults (10 females).Age = 26.5 y, SD = 8.1.BMI = 22.9 kg/m^2^, SD = 3.0.Exclusions *N* = 4 (*N* = 2 did not like test foods, *N* = 1 did not complete all sessions, *N* = 1 refused to eat in 1 session)	Variety manipulated across courses; test meal included fruit only (serving = 200 g).Variety: Given their 4 highest-rated fruits in 4 separate courses (grapes, pineapple, orange, apple, peaches, cantaloupe).Control: Given their highest-rated fruit in each of 4 courses (grapes, pineapple, orange, apple, peaches, or cantaloupe).Outcome: Grams (weighed). Kcal consumed also reported	There was no significant effect of variety condition on weight or energy consumed	None reported	There was a significant interaction reported for course × variety. Intake in course 4 was significantly greater in the variety condition compared with control. Intake in courses 2, 3, and 4 was significantly lower than intake in course 1 in the control condition. Intake in course 4 (grams only) and intake in courses 3 and 4 (energy only) were significantly lower than intake in course 1 in the variety condition	No sample size calculation; control procedures included instructions to eat a meal bar (220 kcal, highest rated for liking in screening) 2 h before the session and to abstain from eating all other foods and energy-containing beverages, sessions were scheduled on separate days (but no more than 1 wk apart), and participants self-reported intake prior to the session to check compliance; pre-post assessment of hunger and fullness in each session reported; pre-post assessment of pleasantness of foods in each session reported; cover story was used, but no indication as to whether this was believed by participants; no additional confounds identified; no additional variables controlled in main model (no significant differences found between sessions in time since eating, food intake prior to sessions, hunger, fullness, or liking)
Roe (2013), US (6)	Field (school); within-subjects; counterbalanced order of conditions using Latin square and randomized assignment of order to classrooms using a computerized randomizer.Sample = 61 children (32 females).Female age = 4.2 y, SEM = 0.1.Male age = 4.6 y, SEM = 0.1.Female BMI percentile = 52.7, SEM = 4.8.Male BMI percentile = 58.5, SEM = 4.6.No exclusions	Variety manipulated within a single course; test meal included fruit and vegetables offered family style (serving = 300 g per bowl) and a pita bread (serving = 16 g).^14^Variety: Each of 3 serving bowls contained a different type of fruit or vegetable (depending on session). If fruits were served, children were offered apple, peach, and pineapple. If vegetables were served, children were offered cucumber, sweet pepper, and tomato.Control: Each of 3 serving bowls contained a single type of vegetable or fruit (depending on session). If a fruit was served, children were offered apple, peach, or pineapple. If a vegetable was served, children were offered cucumber, sweet pepper, or tomato.Outcome: Piece count (number of pieces of vegetables or fruit selected, and any leftovers were recorded by two observers)	There was a significant effect of variety; children consumed more pieces of fruit and vegetables in the variety condition compared with control	None reported	No significant snack type (fruit compared with vegetable) × variety interaction	No sample size calculation; control procedures included a washout period between conditions, conducting each session at a standard time after a nap or quiet play in usual setting; no assessment of appetite reported; assessment of liking of foods was conducted 1 wk after the final session; no information about cover story (although it is reported that adult helpers were unaware of study hypotheses); food intake assessed in groups (social setting is a potential confound, although adult helpers instructed not to influence food intake); food intake assessed using piece count (a potential confound); effects of age, sex-specific BMI-for-age percentile, and food liking on intake were tested as potential covariates; a random effect was included in models to account for the correlation between repeated observations for each child
Roemmich (2010), US (49)	Laboratory; within-subjects; counterbalanced order of conditions, but no method given.Sample = 38 children (19 pairs of male siblings, grouped by weight).Lean-weight sibling age = 12.8 y, SD = 2.5.Overweight sibling age = 12.4 y, SD = 2.1.Lean-weight sibling BMI *z* score = 12.4, SD = 2.1.Overweight sibling BMI *z* score = 1.6, SD = 0.4.No exclusions	Variety manipulated within a single course; test meal included snack assortment (variety meal serving = ∼880 g, control meal serving = ∼800 g).Variety: Served pizza, chicken nuggets, Doritos, iced fruit and oatmeal bites, Skittles, chocolate chip cookies.Control: Served pizza only.Outcome: Grams (weighed). Kcal consumed also reported	Significant effect of variety; children consumed greater energy and weight of food in the variety condition compared with control	None reported	No significant weight classification × variety interaction for intake	No sample size calculation; control procedures included a washout period between conditions, instructions to abstain from eating for 4 h prior to the test session, instructions to avoid all test foods for 24 h prior to session, physical activity was monitored during the week between visits, and test sessions were scheduled during normal dinner hours; baseline assessment of hunger in each session reported; food liking assessed before main study (inclusion criteria specified that participants must report >50-mm liking scores for 5 of 6 test foods); cover story was used, and participant awareness was checked; order of tasks was unclear (timing of eating behavior questionnaires); “sibling” and “family” treated as random effects in models, and presentation order of conditions was considered a potential covariate (but not significant)
Rolls (1981), UK [study 1] (1)	Laboratory; within-subjects; counterbalanced order of conditions, but method unclear. Sample = 36 students (females only).Age range = 18–25 y.BMI range = 18.4–26.4 kg/m^2^.No exclusions	Variety manipulated across courses; test meal included an assortment of sandwiches (serving size unknown).Variety: Given sandwiches with a different filling for each of 4 courses: egg, tomato, cheese, and ham.Control: Given sandwiches with the same filling for each of 4 courses: egg, tomato, cheese, or ham.Outcome: Piece count (number of pieces of sandwich eaten, converted to grams consumed based on average weight of sandwich)	Significant effect of variety; total food intake across courses was greater in the variety condition compared with control	Significant differences in intake for individual courses; intake declined more across courses in the control condition compared with the variety condition. In the variety condition, intake in each course (courses 2, 3, and 4) was greater than intake in the control condition	No significant interaction for time × variety. There were no significant differences in normalized intakes between conditions for “obese” and “leaner” participants	No sample size calculation; control procedures included a washout period between conditions, a standardized mealtime across participants, and instructions to abstain from eating and drinking between a coffee break and the test session at lunch; pre-post assessment of hunger in each session reported; participants ranked liking of foods in a final debriefing session and rated pleasantness before eating (as part of a cover story); cover story was used, and participant awareness was checked in a debrief session; food intake assessed using piece count (a potential confound); no additional variables included in models (no significant differences in intake between subjects who had their favorite sandwich and those who had their least favorite sandwich in the control condition)
Rolls (1981), UK [study 2] (1)	Laboratory; within-subjects; counterbalanced order of conditions, but method unclear. Sample = 24 adults (12 females).Age range: 18–35 y.BMI range: 19.9–24.4 kg/m^2^.No exclusions	Variety manipulated across courses; test meal included an assortment of yogurts (serving = 400–500 g per course).Variety: Given a different yogurt for each of 3 courses: hazelnut, blackcurrant, and orange.Control: Given the same yogurt for each of 3 courses: hazelnut, blackcurrant, or orange.Outcome: Grams (weighed). Energy consumed also reported (unit unclear)	Significant effect of variety (for both grams and energy consumed); total food intake across courses was greater in the variety condition compared with control (averaged across 3 control conditions with 1 yogurt)	Significant differences in intake for individual courses; intake declined more in courses 2 and 3 in the control condition compared with the variety condition. In the variety condition, intake in course 3 was greater than intake in course 3 in the control condition	No significant interaction for time × variety. Intake in each condition was compared for sex, obesity index, yogurt preference ranking, and restraint score; intake was significantly greater for females in the variety condition compared with control, and intake in the variety condition was significantly greater than intake in the control condition with participants’ preferred yogurt. Obesity index and restraint did not significantly influence the effect of variety on intake	No sample size calculation; control procedures included a washout period between conditions and instructions to abstain from eating and drinking between a coffee break and the test session at lunch; pre-post assessment of hunger in each session reported; participants ranked liking of foods in a final debriefing session and rated pleasantness before eating (as part of a cover story); cover story was used, and awareness was checked in a debrief session; no additional confounds identified; no additional variables were included in models
Rolls (1981), UK [study 3] (1)	Laboratory; within-subjects; counterbalanced order of conditions, but method unclear. Sample = 24 students (females only).Age range = 18–20 y.BMI = not reported, but all participants were within 15% of “recommended” body weights.No exclusions	Variety manipulated across courses; test meal included an assortment of yogurts (serving = 400–500 g per course).Variety: Given a yogurt for each of 3 courses that differed only in flavor: cherry, raspberry, and strawberry.Control: Given the same yogurt for each of 3 courses: cherry, raspberry, or strawberry.Outcome: Grams (weighed)	No significant effect of variety for total food intake across courses	No significant differences in intake for individual courses and no significant differences in the rate of decline in intake between conditions	Intake in each condition was compared for restraint scores and obesity; no significant associations were found	No sample size calculation; control procedures included a washout period between conditions and instructions to abstain from eating and drinking between a coffee break and the test session at lunch; pre-post assessment of hunger in each session reported; participants ranked liking of foods in a final debriefing session and rated pleasantness before eating (as part of a cover story); cover story was used, and awareness was checked in a debrief session; no additional confounds identified; no additional variables were included in models
Rolls (1982), UK [study 2]^15^ (2)	Laboratory; within-subjects; counterbalanced order of conditions, but method unclear. Sample = 24 students (12 females).Age range = 18–25 y.BMI range = 18.5–28.9 kg/m^2^.No exclusions	Variety manipulated across courses; test meal included pasta with sauce (serving = 300 g per course).Variety: Given different shapes of pasta for each course: spaghetti, bowtie shapes, and hoop shapes.Control: Given their most preferred shape of pasta in every course (as rated during an initial assessment): spaghetti, bowtie shapes, or hoop shapes.Outcome: Eating intake (amounts of dry pasta and sauce eaten calculated from proportion of pasta to sauce served before meal), converted to energy consumed (kJ)	Significant effect of variety for total food intake across courses; participants consumed more energy in the variety condition compared with control	Significant differences in intake for individual courses; intake was significantly greater in the third course in the variety condition compared with control	No significant interaction for time × variety. Normalized intakes in each condition were compared for sex, restraint scores, and BMI; females and participants who were categorized as more restrained had greater intake in the variety condition compared with control	No sample size calculation; control procedures included a washout period between conditions, instructions to arrive in the same state of hunger on each test day, and meals given at usual time for test foods to be eaten; baseline assessment of hunger in each session reported; pre-post assessment of pleasantness of test foods in each session reported; cover story was used, and awareness was checked in a debrief session; no additional confounds identified; no additional variables were included in models
Rolls (1982), UK [study 3] (2)	Laboratory; within-subjects; counterbalanced order of conditions but method unclear. Sample = 24 students (12 females).Age range = 19–25 y.BMI range = 18.2–32.9 kg/m^2^.No exclusions	Variety manipulated across courses; test meal included an assortment of sandwiches (serving = 200 g per course).Variety: Given sandwiches with a cream cheese filling that had a different flavor in each course: salt flavor, lemon and saccharin flavor, and curry flavor.Control: Given sandwiches with their most preferred cream cheese filling in every course (as rated during an initial assessment): salt flavor, lemon and saccharin flavor, or curry flavor.Outcome: Eating intake (amount eaten in grams calculated from proportion of bread to filling in each piece and number of pieces consumed), converted to energy consumed (kJ)	Significant effect of variety for total food intake across courses; participants consumed more energy in the variety condition compared with control	Significant differences in intake for individual courses; intake was significantly greater in the second and third courses in the variety condition compared with control	No significant interaction for time × variety. Normalized intakes in each condition were compared for sex, restraint scores, BMI, and initial liking of test foods. There was no difference in intake between conditions for participants with initial liking differences of foods of >35 mm, but intake was greater in the variety condition compared with control for participants with initial liking differences of foods of <35 mm. No significant differences were found for sex, restraint scores, or BMI	No sample size calculation; control procedures included a washout period between conditions, instructions to arrive in the same state of hunger on each test day, and meals given at usual time for test foods to be eaten; baseline assessment of hunger in each session reported; pre-post assessment of pleasantness of test foods in each session; cover story was used, and awareness was checked in a debrief session; no additional confounds identified; no additional variables were included in models
Spiegel (1990), US^16^ (50)	Laboratory; within-subjects; counterbalanced order of conditions using a method of alternation. Sample = 27 adults (females only) (participants were grouped by weight).Underweight, age = 24.7 y.Lean weight, age = 31.8 y.Overweight, age = 31.8 y.Underweight, % ideal body weight = 80.1.Lean weight, % ideal body weight = 94.2.Overweight, % ideal body weight = 128.0. No exclusions	Variety manipulated across courses; test meal included an assortment of sandwiches (serving size unknown).Variety, sequential: Given bite-sized sandwiches with 1 of their 3 most preferred fillings in each of 3 courses: tuna, turkey, beef, egg, ham, cheese.Variety, simultaneous: Given bite-sized sandwiches with all 3 of their most preferred fillings in each of 3 courses: tuna, turkey, beef, egg, ham, cheese.Control: Given bite-sized sandwiches with their middle-preferred filling in all 3 courses (as rated during an initial assessment): tuna, turkey, beef, egg, ham, or cheese.Outcome: Piece count (number of pieces of bite-sized sandwiches eaten)	Significant effect of variety for total food intake across courses; for all participants, intake was significantly greater in the simultaneous variety condition compared with control. Intake in the sequential variety condition was not significantly different from intake in the simultaneous or control conditions	Significant differences in intake for individual courses; for all participants, intake was significantly greater in the first and second courses in the simultaneous variety condition compared with control; for all participants, intake was greater in the sequential variety condition compared with the control, but only in the second course	When effects were explored for each weight group separately, differences between conditions for total food intake were only significant for lean weight and overweight participants (intake was significantly greater in the simultaneous variety condition compared with control). Differences between conditions for first and second courses were only significant for lean-weight and overweight participants (intake in the first course was significantly greater in the simultaneous variety condition compared with control for both overweight and lean-weight participants, and intake in the second course was significantly greater in the simultaneous variety condition compared with control for lean-weight participants only)	No sample size calculation; control procedures included a washout period between conditions and instructions to eat the same breakfast at the same time (or skip breakfast) on the morning of each test day, to otherwise abstain from eating until the test session, and food intake prior to the session was checked on arrival; assessment of hunger at the beginning and end of each course reported; pre-post assessment of liking of test foods in each session (at the start and end of the meal) reported; cover story was used, but no awareness check was reported; food intake assessed using piece count (a potential confound); no additional variables were included in models
Stubbs (2001), UK^17^ (51)	Laboratory (Resident trial); within-subjects; randomized order of conditions but no method given. Sample = 12 adults (males only) (participants were grouped by weight).Lean, age = 27.0 y, SD = 2.9.Overweight, age = 39.7 y, SD = 2.9.Lean, BMI = 23.6 kg/m^2^, SD = 1.1.Overweight, BMI = 28.1 kg/m^2^, SD = 0.5.No exclusions	Variety manipulated across meals; choices available for breakfast (serving = 600 g), lunch (serving = 400 g), dinner (serving = 400 g), and snacks (standard portions available for each meal and energy per meal matched across conditions) were manipulated. Cereals were served at breakfast, and a choice of hot dishes and garnish were served at lunch and dinner. Snacks included a mix of soups, desserts, and energy-containing beverages. Tea, coffee, salt, and pepper were available ad libitum at meals across conditions.High variety: Three choices available at breakfast, lunch, and dinner. Six choices of snacks available per day.Medium variety: Two choices available at breakfast, lunch, and dinner. Four choices of snacks available per day.Control: One choice available at breakfast, lunch, and dinner. Two choices of snacks available per day.Outcome: Grams (leftovers collected and weighed). Energy consumed (MJ) also reported	Significant effect of variety for total daily food intake (g and MJ); for all participants, average daily food intake was significantly greater in the high-variety condition compared with the medium-variety and control conditions. Total intake across days is not reported	Differences in intake for each individual meal or for each individual meal session (i.e., breakfast, lunch, dinner) are not reported	Significant weight × variety interaction; lean participants consumed significantly more food in the high-variety condition compared with the medium-variety and control conditions, but there were no significant differences in intake between conditions for overweight participants	No sample size calculation; control procedures included a washout period between conditions, a maintenance diet for 2 d before the trial, instructions to maintain their normal activity routine during the whole study, instructions to abstain from drinking alcohol during the study period, and all participants kept a food log to check compliance with study instructions; hourly assessment of hunger, fullness, and desire to eat on each test day reported; assessment of pleasantness of test foods after each meal reported; no cover story was used; participants were weighed before eating and multiple times throughout the trial (a potential confound); “subject,” “run,” “day,” and “time” were included in models
Vadiveloo (2019), US^18^ (52)	Laboratory; between-subjects; blocked randomization to conditions using ID number.Sample = 184 adults (31.7% female).Age = 34.8 y, SD = 14.1.BMI = 24.4 kg/m^2^, SD = 5.2.Exclusions *N* = 20 (participants included in pretest only)	Variety manipulated within a single course; test meal included fruit and vegetables only. Pears and peppers were served in separate bowls, and depending on condition, different shapes and/or colors of each food were presented in the same bowl (pear serving = 224 g, pepper serving = 140 g).Combination variety: Two conditions (separate groups of participants) were combined to form a single condition. Participants were initially randomly allocated to receive 2 shapes of pears and peppers and to receive both 2 shapes and 2 colors of pears and peppers.Color variety: Participants received 2 colors of pears and peppers (all in the same shape).Control: Participants received a single color and shape of pears and peppers.Outcome: Ounces (weighed)	No significant effect of variety for intake of pears or peppers	None reported	No significant prime × variety, sex × variety, or usual fruit and vegetable intake × variety interactions. Subgroup analyses revealed significant effects of variety when participants were grouped by age and weight status. Participants classified as overweight (using self-reported BMI) had a greater intake of pears in the color variety condition compared with participants classified as having a lean weight or compared with the combination condition, and participants older than 36 y had a greater intake of peppers in the color variety condition than participants younger than 36 y. Participants older than 36 y also had a greater intake of peppers in the color variety condition compared with the combination variety condition	Target sample *N* = 600 reported (Cohen's *d* effect size = 0.31, 1 − β = 0.80, α = 0.05); no control procedures reported; no assessment of appetite reported; assessment of liking reported during questionnaire; cover story was used, but no awareness check was reported; no additional confounds identified; age, weight status, sex, prime, usual fruit and vegetable intake, and presentation order were additional variables included in models
Van Wymelbeke (2020), France^19^ (53)	Field (nursing home); within-subjects; order of conditions was counterbalanced across nursing homes, but allocation method is unclear.Sample = 89 older adults (63 females). Age = 87.5 y, SEM = 0.8.BMI = Not reported. Exclusions *N* = 7 (*N* = 4 participants withdrew from study, and *N* = 3 participants did not complete at least 4 test sessions)	Variety manipulated within a single course; main dish was manipulated and served as part of a multicourse meal consisting of a starter (grated carrots or vegetable soup depending on preference), dessert (custard), and a course of bread with cheese (serving = 810 kcal per whole-meal serving in the variety and control conditions). Participants who did not like cheese (*N* = 11) or custard (*N* = 6) were instead served yogurt and apple puree.Variety: Participants served veal blanquette and 2 garnishes (steamed potatoes and green beans) for main course. Participants were also able to help themselves to 7 condiments (butter, fresh cream, mustard, mayonnaise, tomato sauce, parsley, lemon) in addition to salt and pepper.Control: Participants served veal blanquette and 1 garnish (steamed potatoes) for main course. Participants were also able to help themselves to salt and pepper, but no additional condiments were available.Outcome: Grams (weighed). Energy consumed (kcal) also reported. Piece count (number of spoons or units consumed) used to measure condiment intake	Significant effect of variety for intake; total food intake (in g and kcal) was greater in the variety condition compared with control	Garnish intake was significantly higher when participants were served 2 garnishes in the variety condition compared with control. Bread intake was significantly higher in the variety condition compared with control	Significant condition × repetition interaction; bread intake was significantly higher in the variety condition compared with control in the second replication of the study, but not the first. Dessert intake was also significantly higher in the variety condition in the second repetition compared with the first. Total energy intake was greater in the variety conditions compared with control, but only in the second replication	Target sample *N* = 77 reported (effect considering average SD of 80 for intake in older adults with contingency for 20% dropouts, 1 − β = 0.90, α = 0.05); control procedures included a washout period between conditions, and presenting the test meal at the standard lunch time in the usual setting for the nursing home; pre-post assessment of hunger reported; liking assessed after meal; no information about a cover story; unclear whether participants were tested in groups (social setting is a potential confound); condition, repetition, nursing homes, hunger, and liking were additional variables included in models
Wijnhoven (2015), Netherlands^20^ (7)	Laboratory; within-subjects; randomized order of conditions, but no method given. Sample = 24 older adults (females only).Age = 84.0 y, SD = 8.0.BMI = 24.8 kg/m^2^, SD = 4.9.Exclusions *N* = 5 (*N* = 1 did not show up to study, *N* = 4 dropped out after first session)	Variety manipulated within a single course: test meal with a meat or fish (150–170 g), starch (225 g), vegetable (225 g), and sauce (28 g) component.Variety: If participants chose to have meat, participants received 3 different vegetables of different colors (beets, cauliflower, French beans), 3 different meats (chicken wrapped in bacon, meatball, breaded pork schnitzel), and 3 different starch components (boiled potatoes, fried duchesse potatoes, fried potato slices) in a single course. If participants chose to have fish, participants received 3 different vegetables of different colors (broccoli, carrots, red cabbage), 3 different fish (cod, tilapia, salmon), and 3 different starch components (boiled potatoes, fried Parisienne potatoes, fried potato slices) in a single course.Control: If participants chose to have meat, participants received one vegetable (French beans), one meat component (chicken wrapped in bacon), and one starch component (mashed potatoes), in a single course. If participants chose to have fish, participants received one vegetable (red cabbage), one fish component (tilapia), and one starch component (mashed potatoes), in a single course.Outcome: Grams (weighed). Energy consumed (kcal) also reported. Intake was averaged across the fish/meat test meals for each condition	Significant effect of variety for intake in both energy and grams; total food intake averaged across meals was greater in the variety condition compared with control	Energy intake for the starch component was significantly higher in the variety condition compared with control. Intake in grams was significantly higher for vegetables in the variety condition compared with control. Comparisons for all other components were not significant	None reported	Target sample *N* = 22 reported (effect exceeding 100 kcal with contingency for 25% dropouts, 1 − β = 0.80, α = 0.05); control procedures included a standardized lunch served to participants 4 h before the test meal, a standardized meal setting and time across sessions, and instructions to otherwise abstain from eating and drinking until the test meal (excluding 500 mL of water, tea, or coffee); pre-post assessment of hunger and fullness reported; liking of test foods reported after eating; no information about a cover story (although it is reported that participants were not informed about and unaware of the study aim); no additional confounds identified; testing period effects were included in models
Zeinstra (2010), Netherlands (54)	Field (restaurant); between-subjects; randomized allocation to conditions by schedule days, but method of randomization is unclear.Sample = 326 children (147 females).Age (across conditions) = 5.2 y, SD = 0.7.“No choice” BMI = 15.5 kg/m^2^, SD = 1.7.“Premeal choice” BMI = 15.3 kg/m^2^, SD = 1.7.“Variety” BMI = 15.2 kg/m^2^, SD = 1.7.Exclusions *N* = 23 (*N* = 14 were not able to schedule an appointment, *N* = 9 canceled appointment due to illness or other reason)	Variety manipulated within a single course; vegetables (serving = 130 g) manipulated within a main meal consisting of a starch (130 g), meat (60 g), and dessert (150 g) component.Variety: Two vegetables included in meal (third and fourth preferred vegetable out of carrots, peas, cauliflower,broccoli, red cabbage, beets, French beans, and spinach).Premeal choice: Offered a choice of 1 of 2 vegetables to be included in the meal before being served (children can choose either their third or fourth preferred vegetable out of carrots, peas, cauliflower, broccoli, red cabbage, beets, French beans, and spinach).Control: Randomly assigned to receive 1 of 2 vegetables in the meal (third or fourth preferred vegetable out of carrots, peas, cauliflower, broccoli, red cabbage, beetroot, French beans, and spinach).Outcome: Grams (weighed)	Not reported	There was no significant effect of variety on intake of vegetables, starchy component, meat, or dessert	Effects of age, neophobia, general vegetable liking, restriction, pressure, monitoring, sex, and trait reactance were explored as potential moderators. Significant moderator effect was reported for trait reactance; in the no-choice control condition, high-reactant children consumed significantly less vegetables than low-reactant children	Target sample *N* = 270 (*N* = 90 per condition) reported (effect considering SD of 36 g for vegetable intake, difference of 15g for vegetable intake between conditions, 1 − β = 0.80, α = 0.05); control procedures included that each session was conducted at children's regular dinner time, parents asked not to feed children 1 h before dinner session, andparents asked not to control or influence child's eating behavior during dinner in the restaurant, and parents completed questionnaire about child's eating habits; no assessment of appetite reported; pre-post assessment of liking of foods was reported; no information about cover story; food intake assessed in social setting with parents, and parent and child intakes were significantly positively correlated for all meal components excluding meat (setting is a potential confound); excluding potential moderators, no additional variables included in main models (differences in sex, restriction, pressure, and monitoring were checked across groups but not significant)

1 Means are reported for sample characteristics, or where possible, the range is given if no other descriptives are reported. For BMI, *z* scores or percentiles were extracted if kg/m^2^ was not reported. DEBQ, The Dutch Eating Behaviour Questionnaire; HED, high energy density; LED, low energy density; TFEQ, Three Factor Eating Questionnaire.

2 Country where the study was conducted.

3 Article also reported condition effects on intake for dinner; as foods provided at dinner were the same across conditions and were not manipulated for variety, information relating to dinner intake (dinner intake alone and lunch and dinner intake combined) was not extracted for this review.

4 Effect of variety is reported separately for males and females.

5 Article also included a pilot study for the manipulation used in the main study, but as food liking was the outcome, information was not extracted for this review.

6 Article also reported intake in response to participants self-serving the control meal, changing the dish name, and changing the décor in the room; information from these conditions was not extracted from the article for this review.

7 Given the number of foods presented in this study across courses of the test meal, for brevity, we report only the vegetables that were manipulated for variety.

8 Article also included study 2; variety was manipulated at home for a duration of 4 wk, but as intake was self-reported, information from these conditions was not extracted from the article for this review.

9 Article also included study 1; variety was manipulated across courses (different color of candy in each of 4 courses) and within a single food assortment (mixed colors of candy in each of 4 courses) and compared with a no-variety control condition (same color of candy in each of 4 courses). However, samples of each color were presented to measure liking between courses 2 and 3 in each condition; as this may disrupt intake, information from this study was not extracted for review.

10 Article reports conflicting information for participant age (different values are reported in text and tables).

11 Article also included study 2; the same ingredients were served separately or as a part of a composite meal (stir-fry or pasta) to manipulate the perception of variety. However, as no comparative condition was included in which variety was reduced (i.e., fewer ingredients), information from this study was not extracted for review.

12 Effect of variety is reported separately for males and females.

13 Article also included study 2; participants consumed a test food in the laboratory and were subsequently asked to rate the pleasantness of either the same or a different food item; as food intake was not an outcome of interest, this study was not included for review.

14 As the pita bread was served before the snack began (i.e., before fruit and vegetables were available to children), this was not included in results for total meal intake.

15 Article also included study 1; effect of variety across courses (different color of candy in each of 4 courses) and within a single food assortment (mixed colors of candy in each of 4 courses) was compared with a no-variety control condition (same color of candy in each of 4 courses) and appeared eligible for this review. However, as samples of each candy were presented and consumed between courses 2 and 3 across conditions (to measure liking), intake from courses was disrupted. For this reason, information from this study was not extracted for review.

16 Article also included a “hidden simultaneous” condition in which participants received a mix of their 3 most-preferred flavors of sandwiches for each of 3 courses in a picnic box (so that the whole assortment was hidden from view); information from this condition was not extracted for review.

17 Given the number of foods presented in this study across conditions, for brevity, we report only the number of foods presented for each condition and a short description of the types of foods used.

18 Article also reported intake in response to a positive, negative, and control prime; information from these conditions was not extracted from the article for this review.

19 Article also included a “quality” condition, in which participants received enhanced main dish and dessert recipes, as well as preferable cheeses/bread, without the addition of added variety; information from this condition was not extracted from the article for this review.

20 Study included older adults with a self-reported poor appetite. However, participants did not otherwise report serious illness and/or eating disorder.

### Included studies for review

**FIGURE 1:**
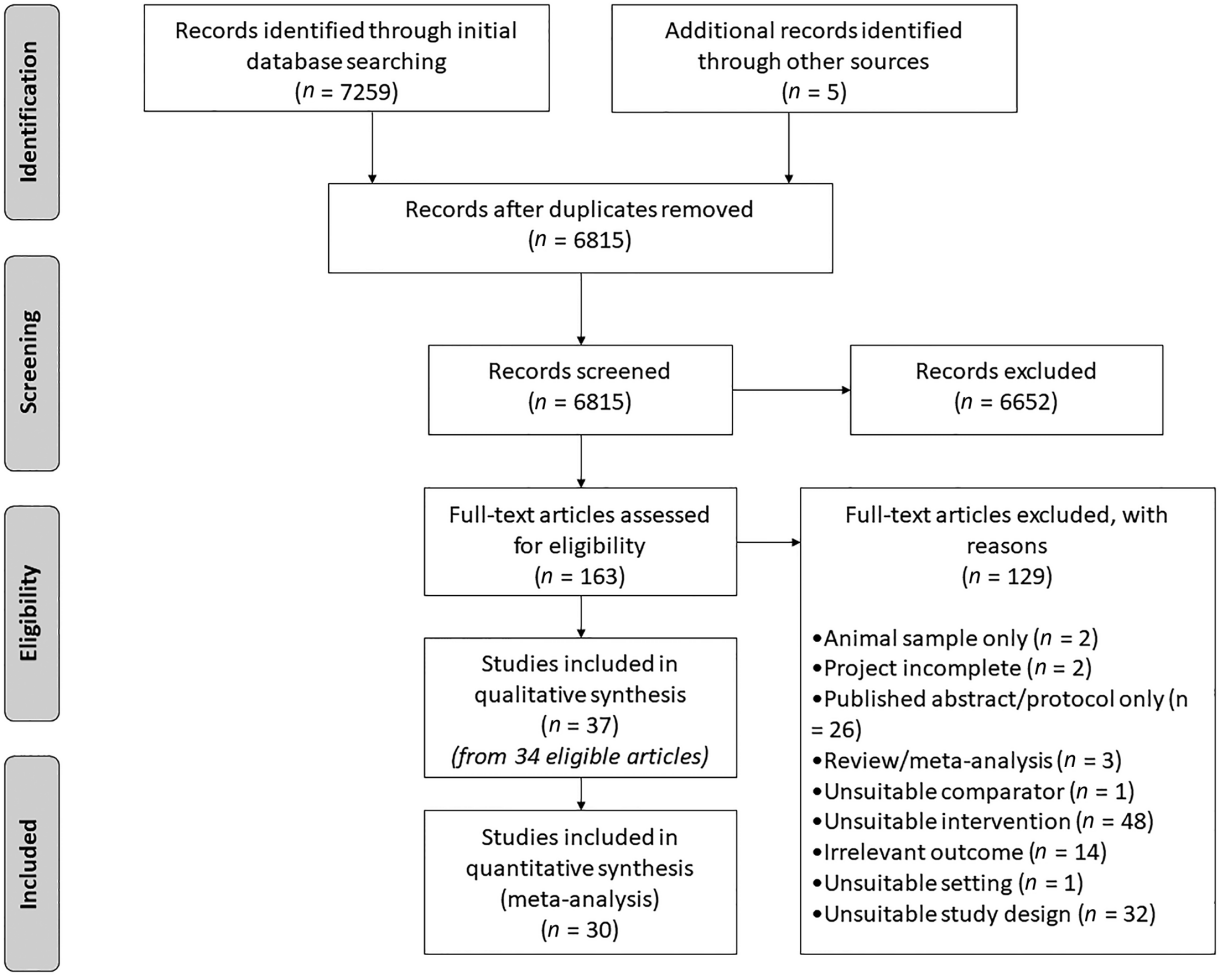
Preferred Reporting Items for Systematic Reviews and Meta-Analysis diagram of the study selection process (55).

An overview of the selection process is presented in **Figure 1**. Database searching identified 7259 articles, of which 29 articles (32 studies) were eligible for the systematic review. No new articles were identified for review in an updated database search. A hand-search identified 5 additional articles for review, consisting of 5 studies.

### Study designs and samples used

Of the 37 studies included for review, 9 were field studies (6, 32, 34, 37–39, 41, 53, 54), and all others were conducted in the laboratory. Twelve studies included variety as a between-subjects factor (4, 19, 21, 22, 39–43, 48, 52, 54), and the remainder were within-subjects. Twelve studies were conducted in the United States (4, 6, 17, 18, 21, 22, 36, 40, 44, 49, 50, 52), 12 in the United Kingdom (1, 2, 20, 32, 33, 35, 37, 46, 51), 5 in the Netherlands (7, 19, 41, 42, 54), 3 in France (38, 47, 53), and a single study was conducted in Denmark (34), Spain (39), Australia (43), Canada (48), and Singapore (45).

Participant age varied across studies; as reported and defined in included articles, 9 studies included children (6, 34, 36, 37, 39–41, 49, 54), 6 included older adults (7, 32, 33, 35, 38, 53), 1 included both children and adults (43), and 1 included both young and older adults (20). All other studies included adults from a community-based (1, 4, 17, 18, 47, 51, 52) or university-based sample (1, 2, 19, 21, 22, 42, 44–46, 48). Seven studies included only females (1, 7, 19, 42, 45, 50), 3 included only males (22, 48, 49), and all others included mixed samples, consisting of an average of 53% females. Studies differed in terms of reporting weight status of participants; although the majority of studies reported participants’ BMI (1, 2, 6, 7, 17–20, 22, 33–35, 37, 41–43, 45–47, 51, 52, 54), 4 studies reported *z* scores (36, 40, 43, 49), 2 studies reported separate height and weight measurements (4, 48), a single study reported percentage ideal body weight (50), and 6 studies included no information on weight status (21, 32, 38, 39, 44, 53). Of those reporting means and variance for BMI, samples had a combined mean of 24.1 kg/m^2^, with a combined SD of 6.8.

### Intervention and outcome measures

Four studies examined the short-term effects of variety across meals on food intake (4, 22, 40, 51). Of these studies, 2 manipulated variety for a main meal dish across 5 d (4, 40), 1 manipulated variety for snacks across 4 d (22), and 1 manipulated variety for breakfast, lunch, evening meals, and snacks across 3 d in a residential trial (51). Three of these studies used a similar design; across conditions, they presented all participants with the same meal or snack on the first and last day of the study, manipulated variety on interval days, and measured pre-post intervention differences in intake (4, 22, 40). The remaining study (51) manipulated variety within each meal session for each day (e.g., foods served for breakfast differed on days 1, 2, and 3) and reported differences in average daily intake across conditions. All 4 studies used a combination of test foods in meals that varied multiple sensory characteristics (i.e., foods differed in appearance, texture, and flavor) across conditions.

The majority of studies examined the short-term effects of variety within meals on food intake. Nine studies manipulated variety across multiple courses of a meal (1, 2, 18, 20, 45, 50). In these studies, the same or a different food was presented for each successive course, intake for each individual course was measured, and cumulative food intake was calculated. One study manipulated variety within a hot lunch meal (2), and another offered participants an assortment of fruit (18). All others presented participants with snack foods: sandwiches with different fillings (1, 2, 20, 50), an assortment of chocolates (45), and an assortment of yogurts (1). In 3 studies, foods varied in a single sensory characteristic (1, 2). All others presented foods that varied in appearance, texture, and flavor.

Similarly, 24 studies manipulated variety within a single course of a meal (6, 7, 17, 19, 21, 32–39, 41–44, 46–49, 52–54). In these studies, the number of different components and/or sensory characteristics present within a single course was manipulated across conditions, and intake was measured. One study manipulated variety for every component in a multicomponent meal and offered 3 different meats (or fish), 3 different vegetables, and 3 different starch foods in the variety condition (7). Nine studies manipulated variety for a single component within a multicomponent meal; 4 studies offered a selection of different vegetables (17, 37, 47, 54), 2 offered a selection of different vegetables/sides and condiments (38, 53), 3 offered additional condiments for a set main meal (32, 33, 35), 1 offered 3 components instead of 2 components within a meal (44), and another offered a single vegetable with different seasonings (36). Four studies manipulated variety within a selection of fruit and/or vegetables that were served alone and without any other components in the meal (6, 34, 39, 52). All other studies manipulated variety within snack foods: savory hors d'oeuvres (48), sandwich fillings (46), ice cream (21), colored candies (19), marshmallows (41), and cookies (42). This includes 2 studies that presented participants with an assortment of sweet and savory snacks (43, 49). In 3 studies, foods varied in a single sensory characteristic (19, 21, 36). All others presented foods that varied in appearance, texture, and flavor.

To measure food intake, the majority of studies weighed servings and leftovers; 10 studies reported weight consumed in grams (1, 4, 7, 19–21, 36, 39, 54), 1 study reported weight consumed in ounces (52), 3 studies reported energy consumed in calories (22, 41, 42), 3 studies reported energy consumed in kilojoules (2, 45), 15 studies reported weight and energy consumed (17, 18, 32, 33, 35, 37, 38, 40, 43, 44, 46, 47, 49, 51, 53), and 1 study reported intake as a percentage (34). Six studies used a piece count to measure intake; 5 studies reported the number of units consumed (6, 38, 48, 50, 53), and 1 study converted number of pieces to grams consumed using average unit weight (1). In 2 studies, all or some foods were eaten outside of a monitored test session, and leftovers were returned to or collected by experimenters the following day (22, 51).

### Other data analyses

In addition to reporting main effects of variety on total food intake, some studies also reported effects on single or multiple components within a meal, interaction effects with other factors that may influence food intake, and subgroup analyses (see Table 2). Additional variables in analyses included impulsivity (19), response inhibition (41), reward sensitivity (41, 42), trait reactance (54), multiple eating behavior traits (2, 45, 46), choice (39, 47), priming effects (52), portion size (37), dishware size (43), consumption of a preload (46), food liking (2, 54), hunger (42), neophobia (54), and propylthiouracil taster status (36). As these variables were included in only 1 or 2 studies and different measures were used, we did not investigate subgroup effects for these variables in the meta-analysis.

### Risk of bias

**FIGURE 2:**
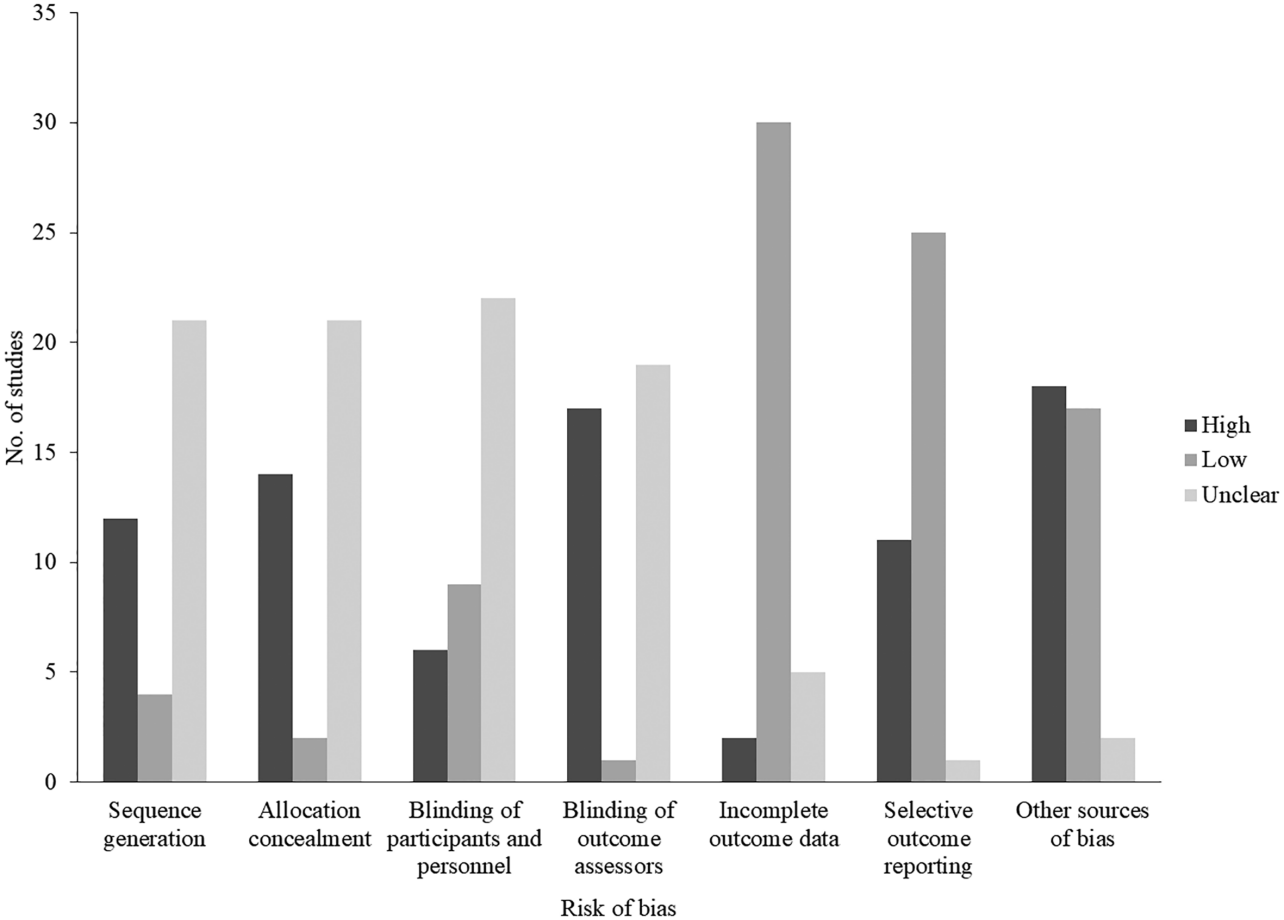
Risk of bias assessment for each criterion across studies (*N* = 37).

Risk of bias was found to be low for the majority of studies when outcome reporting and control of confounding variables were assessed; 30 studies specified their exclusion criteria and justified data exclusions (1, 2, 6, 7, 17, 18, 20, 22, 32, 33, 35–38, 40–47, 49–53), 25 studies fully reported all prespecified outcomes of interest in line with their reported data analysis plan (2, 6, 7, 17, 18, 20, 22, 32, 33, 35–38, 40, 43, 45–47, 49–54), and no confounding variables were identified in 17 studies (1, 2, 7, 17–20, 22, 33, 36, 40–42, 46, 47). However, for remaining criteria relating to the awareness of study outcomes and assignment to conditions, risk was found to be high or unclear for most studies. Only 4 studies reported the use of a random component to allocate participants to conditions or counterbalance condition order (studies were deemed to be “high risk” if they counterbalanced condition order but reported a method that relied on alternation or “unclear” if no method was reported) (6, 41, 43, 52), 2 studies reported a method that adequately concealed condition allocation before and during enrollment (6, 43), 9 studies used a cover story and assessed participant beliefs accordingly (1, 2, 41–43, 49), and 1 study reported that the experimenter who assessed food intake was blind to the study aims or conditions (43). See **Figure 2** for an overview of results for each criterion.

In addition, we found that 3 studies reported no procedures to control appetite before the test session (39, 41, 52), 10 studies reported no assessment of appetite on the test day (4, 6, 19, 21, 34, 37, 39, 44, 52, 54), and 6 studies reported no assessment of liking for test foods (19, 21, 41–44). Sample sizes were often small, with 17 studies including <30 participants per condition (1, 2, 4, 7, 18, 20–22, 32, 35, 40, 44, 45, 50, 51). Six studies estimated their required sample size prior to data collection (7, 37, 46, 52–54), but only 4 met their target sample (7, 46, 53, 54).

### Overview of main results of studies

Of 37 studies included in the review, 16 reported a significant effect where variety increased food intake (1, 2, 4, 6, 7, 20, 39, 40, 44, 46, 48, 49, 51, 53). No significant effect was found in 6 studies (1, 19, 22, 36, 47, 54), and findings were mixed in 15 studies (e.g., significance and/or direction of effect differed depending on the unit of measurement reported for intake, individual components for which intake was assessed, or the inclusion of moderating factors in models) (17, 18, 21, 32–35, 37, 38, 41–43, 45, 50, 52).

### Meta-analysis

Of 37 studies reviewed, data from 30 studies (consisting of 39 comparisons) were used in the meta-analysis for the main effect of variety on total meal intake. This included 3 studies reporting pre-post data for variety and control conditions (4, 22, 40). Data from 2 studies were excluded from the meta-analysis, as articles did not provide sufficient information to calculate effect size (42, 48). Four studies were excluded as they did not report effects on total meal intake; 1 study reported average daily food intake but did not report intake for individual meals or meal sessions across days (51), and 3 studies reported intake for only single components within a multicomponent meal (and thus did not report effects on total meal intake) (18, 37, 54). One study was identified as an outlier and excluded from analyses; it was the only study to report separate comparisons for restrained (Hedges' *g* = –19.500, *P* < 0.001) and unrestrained eaters (Hedges' *g* = 28.292, *P* < 0.001), and effect sizes were noticeably different from all other studies included in the meta-analysis.

**FIGURE 3:**
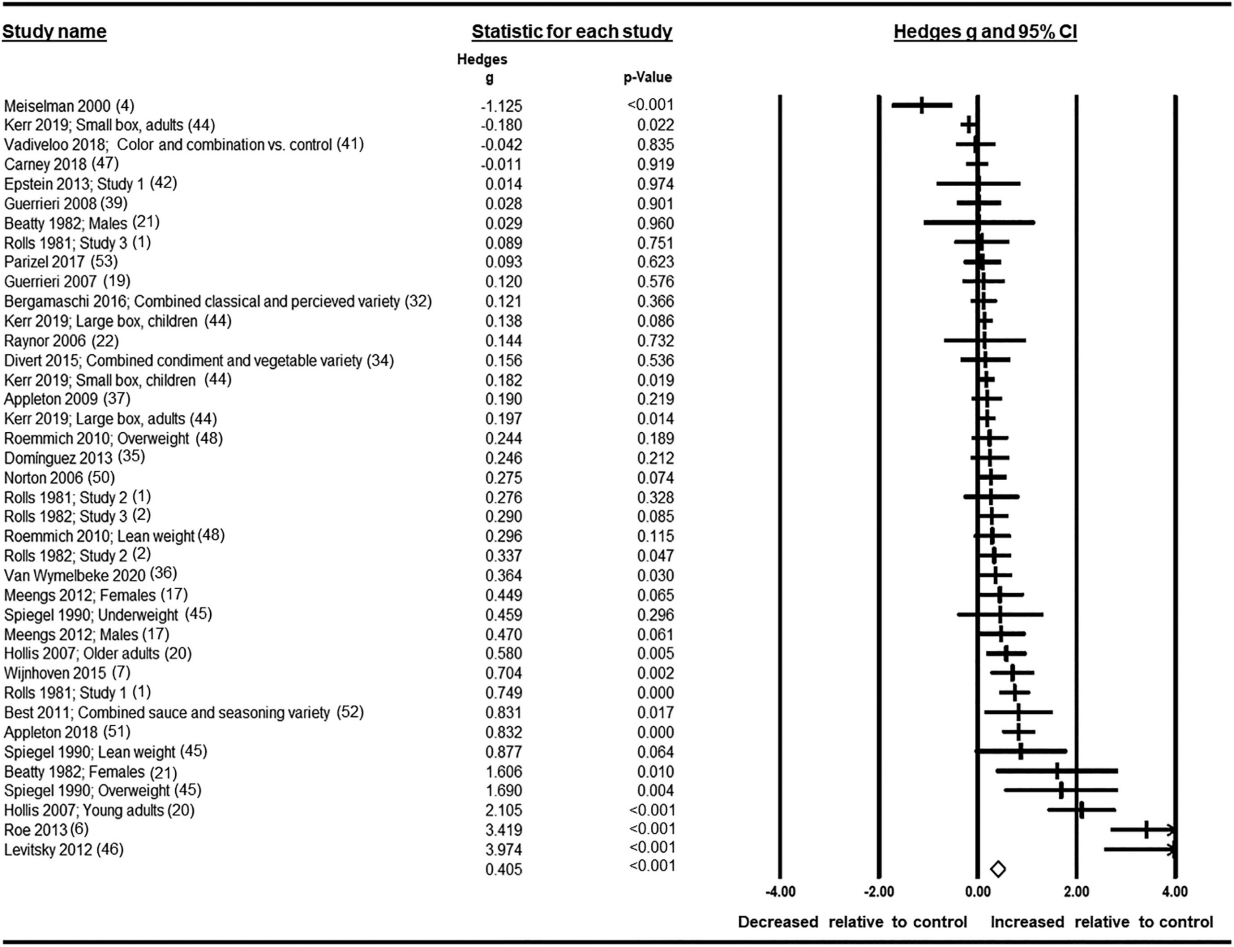
Forest plot displaying effect sizes for comparisons from each study included in the meta-analysis (*N* = 30).

The meta-analysis reported a significant small to medium effect size of variety on total meal intake (in weight and energy) (Hedges' *g* = 0.405; 95% CI: 0.259, 0.552; *z* = 5.413; *P* < 0.001). Sensitivity analyses using the “1 study removed” procedure revealed no change in results, and the overall effect size remained within the confidence interval and was significant after adjusting the imputed correlation value (*r* = 0.2, Hedges' *g* = 0.398, *P* < 0.001; *r* = 0.9, Hedges' *g* = 0.402, *P* < 0.001). This strengthens evidence that the overall effect size is within the specified range, irrespective of imputed values, and favors increased intake in the presence of variety. However, there was considerable heterogeneity between comparisons (*I*^2^ = 84%). See **Figure 3** for a forest plot.

### Planned subgroup analyses

**Table 3** displays effect sizes and *P* values for planned subgroup analyses, including effects at each subgroup level. To identify sources of heterogeneity across studies and explore effects of potential moderators, we assessed differences in study manipulations and sample characteristics. There were no significant between-subgroup effects when studies were categorized by the form of variety manipulated (across meals compared with between courses compared with within a single course), the test foods used (fruit and vegetables compared with other food groups), or the number of sensory characteristics varied in the experimental condition (single compared with multiple). When investigating differences in effects for test foods, comparisons were excluded from the analysis if studies manipulated variety for both food categories (4, 7, 53). Notably, when examining subgroup effects for test foods and the number of sensory characteristics manipulated, removing studies in which a combination of different foods was presented within a single course (17, 32, 33, 35, 36, 38, 47) did not affect results. There were also no significant between-subgroup effects when studies were categorized by participant age (children compared with adults compared with older adults), sex (male compared with female), or body weight (underweight compared with lean compared with overweight). No heterogeneity was observed within the subgroup for males or underweight, and heterogeneity was low within the subgroup for lean (*I*^2^ = 23%), moderate for studies manipulating a single sensory characteristic (*I*^2^ = 38%), and substantial for older adults (*I*^2^ = 52%), females (*I*^2^ = 51%), and studies manipulating variety between courses (*I*^2^ = 73%). Heterogeneity within all other subgroups was considerable (*I*^2^ > 75%).

**TABLE 3:** Effect sizes and *P* values for planned subgroup analyses

Variable	Subgroup level (*N* comparisons)	Effect size, Hedges' *g* (95% CI)^1^	*P* for total between-subgroup heterogeneity^2^	*Q*
Form of variety	Across meals (*N* = 3)	–0.363 (–1.224, 0.498)	0.062 (0.372)	5.575
	Between courses (*N* = 10)	0.651 (0.342, 0.960)***		
	Within a course (*N* = 26)	0.377 (0.215, 0.539)***		
Test foods	Fruits & vegetables (*N* = 8)	0.516 (0.069, 0.963)*	0.689 (4.134)	0.160
	Other food groups (*N* = 27)	0.419 (0.260, 0.578)***		
No. sensory components	Multiple (*N* = 32)	0.447 (0.277, 0.618)***	0.059 (0.354)	3.562
	Single (*N* = 7)	0.192 (–0.012, 0.395)		
Age	Children (*N* = 10)	0.364 (0.090, 0.639)**	0.715 (4.290)	0.672
	Adults (*N* = 22)	0.407 (0.182, 0.632)***		
	Older adults (*N* = 7)	0.501 (0.285, 0.718)***		
Sex	Male (*N* = 5)	0.291 (0.074, 0.507)**	0.101 (0.606)	2.691
	Female (*N* = 9)	0.584 (0.308, 0.860)***		
Body weight	Overweight (*N* = 2)	0.861 (–0.540, 2.263)	0.845 (5.070)	0.337
	Lean (*N* = 2)	0.424 (–0.049, 0.896)		
	Underweight (*N* = 1)	0.459 (–0.402, 1.320)		

1 Effect size reported for each subgroup level; asterisk indicates statistical significance (*P < 0.05, **P < 0.01, ***P < 0.001).

2 *P* value reported for mixed-effects analysis; Bonferroni-corrected *P* included in brackets.

## Discussion

To our knowledge, this is the first systematic review and meta-analysis to formally synthesize experimental studies and quantify the size of the effect of variety on total food intake within and across meals. In line with past narrative reviews (14–16), we found evidence of an overall effect of variety that favored increased meal intake. Of 37 studies included in the review, 16 reported a significant effect in which variety increased food intake (1, 2, 4, 6, 7, 20, 39, 40, 44, 46, 48, 49, 51, 53), 6 reported no significant effect (1, 19, 22, 36, 47, 54), and 15 reported mixed findings (17, 18, 21, 32–35, 37, 38, 41–43, 45, 50, 52). A meta-analysis of data from 30 studies showed that variety had a small to medium effect on meal intake. This overall effect appears to be robust, as sensitivity analyses showed no change in results.

It is generally believed that sensory-specific satiety is the mechanism by which variety influences food intake (12, 13, 56). Sensory-specific satiety has been shown to occur across age groups (20, 57), although it appears to be less pronounced in older adults (20, 58). It also occurs irrespective of sex (16) and weight status (48, 50, 59, 60), thus mirroring the effects of variety on food intake between subgroups in this meta-analysis.

It is clear that variety is a key driver of food intake across the life span and may be of interest to the development of cost-effective, public health interventions to improve population well-being, as variety can be related to better nutrition and diet quality (5). For instance, studies included in this review that focused on children tended to explore how variety can be used to increase intake of fruit and vegetables (6, 34, 36, 37, 39, 54). Several studies also aimed to increase food intake in older adults in the presence of variety (7, 32, 33, 35, 38, 53), as this age group is known to be at greater risk of undernutrition due to poor appetite (61, 62). However, results of this review suggest that interventions may be premature; only 2 studies exploring effects in children reported a significant increase in intake for fruit and vegetables in the presence of variety (6, 39), while remaining studies reported mixed (34, 37) or nonsignificant results (36, 54). For older adults, 2 studies reported a significant increase in food intake in the presence of variety (7, 53), while remaining studies reported mixed results (32, 33, 35, 38). Further evidence is needed to identify a consistent effect of variety in these contexts.

Results also suggest that dietary strategies focused on variety could have important implications for the management of energy intake and, in turn, weight control. Although they did not meet inclusion criteria to be eligible for this review, some intervention trials have shown that assigning individuals to a long-term diet that limits variety for high energy-dense foods can reduce energy derived from these foods (63–65), decrease hedonic ratings for these foods (64, 65), and encourage greater weight loss to occur (63). A previous commentary has highlighted that such dietary treatments should simultaneously encourage an increase in variety for more nutritious foods in order to promote consistent effects on weight loss and weight maintenance (66). Again, we note that results appear to differ across studies measuring effects of variety for fruits and vegetables on meal intake in both children and adults, as wide confidence intervals indicate instability in the effect size estimate. As such, in line with Vadiveloo and Parekh's recommendation (66), we emphasize a need for future research to examine long-term effects, as the influence of variety for fruits and vegetables may be more apparent when a need to “compensate” for reduced energy derived from high energy-dense foods is considered in a treatment setting.

Considerable heterogeneity present across studies was not explained by subgroup analyses; there were no significant differences in effect size when studies were categorized by the form of variety manipulated, the test foods used, the number of sensory characteristics varied, or key demographics of samples. However, clear methodologic differences between studies were identified in the review (see Table 2). For instance, the difference in variety between experimental and control conditions was smaller within some studies compared with others, particularly when the control condition consisted of multiple foods and sensory characteristics in and of itself. It may be that the effects of variety were undermined in such comparisons, and the question of whether there is a ceiling effect at which point further increases in variety no longer result in increased intake warrants further exploration in future research. Studies were also often designed to manipulate multiple factors in addition to variety, and different variables were controlled in models across studies. Given that studies often reported the use of small samples, it is likely that more than half of studies were at risk of being underpowered. Risk of bias was also deemed to be high or unclear for most studies when procedures for sequence generation, allocation concealment, and blinding were assessed. In order to confirm our results, there is a clear need to conduct well-powered, blinded, randomized control studies that are specifically focused on variety.

Considering the limitations of studies included in this systematic review and meta-analysis, we put forward 4 methodologic recommendations for future research as they apply to the topic of food variety, although we acknowledge applications to the wider literature of human appetite research (67). First, it would be useful for studies to report a priori sample size calculations to distinguish between planned and exploratory analyses and to consider the impact of exploring effects of multiple factors and comparisons on study power. Second, we emphasize a need to develop a consistent definition of variety that also considers the number of components that constitute variety, the possibility of a point of saturation for variety within a meal, and possible effects of variety in a control condition that may affect effect size [for further discussion on defining variety, see (14)]. Third, it would be useful to develop clear guidelines for best practice regarding the design of future studies and better reporting of methods and results. Finally, we encourage researchers to adopt open science practices in order to facilitate in the future synthesis of studies in this field, such as preregistration and open access data sets (68).

Limitations of this systematic review and meta-analysis should also be acknowledged. Although efforts were made to ensure that data extraction was accurate and consistent across articles, the use of single data extraction can have a higher error rate than double data extraction (69). In order to synthesize studies, we focused on the short-term main effect of variety on total meal intake at the exclusion of analyses within studies that explored additional effects of variety on food intake. For instance, it should be highlighted that some studies reported significant effects of variety on intake for individual components of a meal (7, 33, 35, 37, 38, 44, 53), conflicting results depending on whether intake was reported in energy or weight (17, 32, 43), and significant interaction or subgroup effects for variety (17, 20, 33, 36, 41–43, 45, 50–54). It should also be acknowledged that only 4 studies eligible for review manipulated variety across meals, meaning effects on intake for variety when meals are repeated and eaten over a longer period remain unclear.

This systematic review and meta-analysis was also limited by the data available. Some studies were excluded from data analyses due to missing information required for inclusion in the meta-analysis. Missing data for the correlation between conditions were estimated for most studies that used a within-subjects design, and a summary effect size was computed for studies reporting multiple comparisons using the same participants to avoid issues associated with multiplicity, although sensitivity analyses suggest that this had a limited influence on results. However, as heterogeneity was high, publication bias could not be reliably assessed. Considering the high number of studies reporting significant results, some evidence of publication bias is likely, and findings of the meta-analysis should be interpreted with caution.

Therefore, although we found evidence to support that food intake is increased in the presence of variety, risk of bias and methods used to measure effects on food intake were a concern, particularly as subgroup analyses could not account for heterogeneity. It is recommended that further attention is given to the development of preregistered, well-powered randomized controlled studies in eating behavior research and to the consideration of variety as a key driver of food intake in dietary interventions.

## Supplementary Material

nqaa352_Supplemental_File

## Data Availability

Data described in the manuscript will be made publicly and freely available without restriction at https://osf.io/ze6cr/.

## Notes

Funding: Supported by the Economic and Social Research Council (ESRC Wales Doctoral Training Partnership) (Project Reference: ES/P00069X/1, Studentship 197139). The funders had no direct role in the study design, data collection, or analysis for this research.

Supplemental Methods 1 and 2 are available from the “Supplementary data” link in the online posting of the article and from the same link in the online table of contents at https://academic.oup.com/ajcn/.
