# Supplementary material for: Effect of food variety on intake of a meal: a systematic review and meta-analysis
Source: Am J Clin Nutr. 2021 Jan 29;113(3):716–41. doi: 10.1093/ajcn/nqaa352 (PMC7948867; doi:10.1093/ajcn/nqaa352)
Supplement: nqaa352_Supplemental_File [file nqaa352_supplemental_file.docx]

**Online Supplementary Material**

**Effect of food variety on intake of a meal: A systematic review and meta-analysis**

Rochelle Embling^1^, Aimee E. Pink^1,2^, Jennifer Gatzemeier^1^, Menna Price^1^, Michelle Lee^1^, Laura L. Wilkinson^1^

^1^Department of Psychology, College of Human and Health Sciences, Swansea University, Swansea, SA2 8PP, UK. Email RE: 825379@swansea.ac.uk

^2^School of Social Sciences, Nanyang Technological University, Singapore, 639818.

Corresponding author: Rochelle Embling, Department of Psychology, College of Human and Health Sciences, Swansea University, Swansea, SA2 8PP, UK. Phone: 01792 29 5279. Email: [825379@swansea.ac.uk](mailto:825379@swansea.ac.uk).

**Description of supplementary materials:**

**Supplementary methods 1** Key terms included in search strategy.

**Supplementary methods 2** Details of criteria used to assess Risk of Bias.

**Online Supplementary Material**

**Supplementary methods 1** Key terms included in search strategy; example provided for use in Web of Science.

(variety[Title] OR varied[Title] OR various[Title] OR “dietary diversity”[Title] OR "sensory variety"[Title] OR "food group*"[Title] OR monoton*[Title])

AND

("energy intake" OR intake[Title] OR consum*[Title] OR portion*[Title] OR serving*[Title] OR “meal size”[Title])

AND

(meal*[Title] OR snack*[Title] OR food*[Title] OR breakfast[Title] OR lunch[Title] OR dinner[Title] OR course*[Title] OR buffet[Title] OR dietary[Title])

Limits: publication date from 1980/01/01, Humans, English

*If possible, additional limits were included to filter by article type (e.g. exclude reviews, commentaries, meta-analyses)

**Online Supplementary Material**

**Supplementary methods 2** Cochrane risk-of-bias tool guidelines^[[1]](#footnote-1)^, modified for use following the approach of Buckland and colleagues^[[2]](#footnote-2)^

1. **Sequence generation**

Describe the method used to generate the allocation sequence in sufficient detail to allow an assessment of whether it should produce comparable groups.

**High** = no random element was used in generating the allocation sequence or the sequence is predictable. Examples include alternation; methods based on dates (of birth or admission); patient record numbers; allocation decisions made by clinicians or participants; allocation based on the availability of the intervention; or any other systematic or haphazard method.

**Low** = If a random component was used in the sequence generation process. Examples include computer-generated random numbers; reference to a random number table; coin tossing; shuffling cards or envelopes; throwing dice; or drawing lots. Minimization is generally implemented with a random element (at least when the scores are equal), so an allocation sequence that is generated using minimization should generally be considered to be random.

**Unclear** = If the only information about randomization methods is a statement that the study is randomized, or no information is given.

**NB.** If between-subjects, this should refer to the allocation of participants to different conditions. If within-subjects, this should refer to the order of conditions (i.e., the use of counterbalancing).

1. **Allocation concealment**

Describe the method used to conceal the allocation sequence in sufficient detail to determine whether intervention allocations could have been foreseen in advance of, during, enrolment.

**High** = If participants or investigators enrolling participants could possibly foresee assignments and thus introduce selection bias, such as allocation based on: using an open random allocation schedule (e.g. a list of random numbers); assignment envelopes were used without appropriate safeguards (e.g. if envelopes were unsealed or non-opaque or not sequentially numbered); alternation or rotation; date of birth; case record number; or other explicitly unconcealed procedures. Also, answer ‘high' if there is reason to suspect that the enrolling investigator or the participant had knowledge of the forthcoming allocation.

**Low** = If the participants and investigators enrolling participants could not foresee assignment because one of the following, or an equivalent method, was used to conceal allocation: central allocation (including telephone, web-based and pharmacy-controlled randomization); sequentially numbered drug containers of identical appearance; or sequentially numbered, opaque, sealed envelopes.

**Unclear** = If no information about allocation concealment is given.

1. **Blinding of participants and personnel**

Performance bias due to knowledge of the allocated interventions by participants during the study. Assessment should be based on whether a cover story was used and believed by participants (following Buckland, Er, Redpath, & Beaulieu, 2018).

**High** = If no cover story is used and/or participants were aware of their allocated condition throughout the study.

**Low** = If a cover story is used and participants report being unaware of their allocated condition throughout the study.

**Unclear** = If no information is given about the use of a cover story. If a cover story is used, no information is given about whether or not this was believed by participants.

1. **Blinding of outcome assessors**

Based on whether the experimenter who assessed food intake was blind to the study aims or condition administered (following Buckland, Er, Redpath, & Beaulieu, 2018).

**High** = If the experimenter who assessed food intake was aware of the study aims or condition administered when presenting foods to participants and/or when calculating food intake (e.g. weighing leftovers).

**Low** = If the experimenter who assessed food intake was blind to the study aims or condition administered when presenting foods to participants and when calculating food intake (e.g. weighing leftovers).

**Unclear** = If no information is given about awareness of conditions, and it is unclear whether the experimenter was aware of study conditions.

1. **Incomplete outcome data**

Based on whether the exclusion of participants was specified in the exclusion criteria or exclusions deviated from standard procedures in the research field (following Buckland, Er, Redpath, & Beaulieu, 2018).

**High** = If exclusion criteria are specified but not justifiable (i.e., they are not standard practice in the field and are not otherwise justified by the researcher). Any exclusion of participants from data analyses are not justified by the researcher.

**Low** = If exclusion criteria are specified and clearly justified by the researcher; criteria are standard practice in the field and otherwise justified for the present study. Any exclusion of participants from data analyses are justified by the researcher.

**Unclear** = If exclusion criteria are not specified.

1. **Selective outcome reporting**

Reporting bias due to selective outcome reporting.

**High** = if not all of the study’s pre-specified primary outcomes have been reported; one or more primary outcomes is reported using measurements, analysis methods or subsets of the data (e.g. subscales) that were not pre-specified and are not identified as exploratory analyses; one or more outcomes of interest in the review are reported incompletely so that they cannot be entered in a meta-analysis; the study report fails to include results for a key outcome that would be expected to have been reported for such a study.

**Low** = if the study protocol/data analysis plan is available and all of the study’s pre-specified (primary and secondary) outcomes that are of interest in the review have been reported in the prespecified way, or if the study protocol is not available but it is clear that the published reports include all expected outcomes, including those that were pre-specified.

**Unclear** = A study protocol/data analysis plan is not included.

1. **Other sources of bias**

Based on risk of confounding variables influencing food intake - e.g. used piece count that is susceptible to researcher bias, absence of procedures to control for appetite between conditions; experiment conducted in a social setting outside of the laboratory (e.g. in a cafeteria); administering psychometric scales related to eating before assessing food intake. Please list confounding variables in textbox.

**High** = At least one confounding variable is identified, and it is not adequately controlled in the experimental procedure.

**Low** = No confounding variables identified, or confounding variables are adequately controlled in the experimental procedure.

**Unclear** = The method is lacking detail to identify whether confounding variables are present (e.g. setting is not described, method for calculating intake is not explained, order of tasks is not clear, sample characteristics not fully reported).

1. Retrieved from Higgins JPT, Sterne JAC, Savović J, Page MJ, Hróbjartsson A, Boutron I, Reeves BC, Eldridge S. A revised tool for assessing risk of bias in randomized trials. Cochrane Methods. *Cochrane Database of Systematic Reviews* 2016 [cited 2020 Jul 13]; 10:52. Available from: <http://doi.wiley.com/10.1002/14651858.CD201601> [↑](#footnote-ref-1)
2. Buckland NJ, Er V, Redpath I, Beaulieu K. Priming food intake with weight control cues: Systematic review with a meta-analysis. International Journal of Behavioral Nutrition and Physical Activity. BioMed Central Ltd.; 2018 [↑](#footnote-ref-2)
